# Intermediate gray matter interneurons in the lumbar spinal cord play a critical and necessary role in coordinated locomotion

**DOI:** 10.1371/journal.pone.0291740

**Published:** 2023-10-31

**Authors:** Naëmi Kuehn, Andreas Schwarz, Carlo Antonio Beretta, Yvonne Schwarte, Francesca Schmitt, Melanie Motsch, Norbert Weidner, Radhika Puttagunta

**Affiliations:** 1 Laboratory for Experimental Neuroregeneration, Spinal Cord Injury Center, Heidelberg University Hospital, Heidelberg, Germany; 2 Laboratory for Experimental Neurorehabilitation, Spinal Cord Injury Center, Heidelberg University Hospital, Heidelberg, Germany; 3 Department of Functional Neuroanatomy, Institute for Anatomy and Cell Biology, Heidelberg University, Heidelberg, Germany; 4 Institute of Pharmacology, Heidelberg University, Heidelberg, Germany; 5 Spinal Cord Injury Center, Heidelberg University Hospital, Heidelberg, Germany; Szegedi Tudomanyegyetem, HUNGARY

## Abstract

Locomotion is a complex task involving excitatory and inhibitory circuitry in spinal gray matter. While genetic knockouts examine the function of individual spinal interneuron (SpIN) subtypes, the phenotype of combined SpIN loss remains to be explored. We modified a kainic acid lesion to damage intermediate gray matter (laminae V-VIII) in the lumbar spinal enlargement (spinal L2-L4) in female rats. A thorough, tailored behavioral evaluation revealed deficits in gross hindlimb function, skilled walking, coordination, balance and gait two weeks post-injury. Using a Random Forest algorithm, we combined these behavioral assessments into a highly predictive binary classification system that strongly correlated with structural deficits in the rostro-caudal axis. Machine-learning quantification confirmed interneuronal damage to laminae V-VIII in spinal L2-L4 correlates with hindlimb dysfunction. White matter alterations and lower motoneuron loss were not observed with this KA lesion. Animals did not regain lost sensorimotor function three months after injury, indicating that natural recovery mechanisms of the spinal cord cannot compensate for loss of laminae V-VIII neurons. As gray matter damage accounts for neurological/walking dysfunction in instances of spinal cord injury affecting the cervical or lumbar enlargement, this research lays the groundwork for new neuroregenerative therapies to replace these lost neuronal pools vital to sensorimotor function.

## Introduction

Interest has been building to better understand the role spinal interneurons (SpINs) play in motor function particularly in the cervical and lumbar enlargements [1–6]. Interconnected inhibitory and excitatory interneurons (INs) form plastic networks within these two spinal enlargements and are responsible for eliciting locomotor output and coordinating sensory and motor function for and between limbs.

It has been previously shown in rats that a large gray matter lesion in the rostral-lumbar enlargement resulted in gross locomotor deficits. Gross hindlimb function was most severely affected when neuronal loss included spinal level L2; these locomotor deficits were not observed in a thoracic gray matter lesion, highlighting the importance of neurons at this spinal level [7, 8]. Furthermore, these deficits did not correlate with motoneuron loss. While it was suggested that the intermediate gray matter plays a crucial role in the development of these deficits, the most severe neuronal damage was seen in both the dorsal horn and intermediate gray matter [7]. Although damage to the dorsal horn is more commonly associated with sensory deficits, it is unclear whether this also played a role in behavioral deficits. Damage to the dorsal horn can evoke pain [9] and animals with pain in their hindlimbs can show altered gait patterns [10, 11]. Therefore, loss of afferent or other information due to the severity of the lesion may have impacted motor output.

Spinal IN (SpIN) ensembles in the intermediate gray matter receive and gate descending motor and ascending sensory input and elicit signals to other INs and motoneurons. Elegant cFos and genetic knockout/silencing experiments have revealed the functions of IN populations in laminae V-VIII in the lumbar cord during locomotion in both mice and rats [2, 12–16]. While these experiments highlight the roles of individual SpIN subtypes, thus far no study has examined the phenotype of combined SpIN loss. Previous studies have shown that changes to the ratio of excitatory to inhibitory INs can influence the speed and pattern of the motor output as well [17]. For example, inhibitory V1 INs have been shown to be necessary for fast motor bursting. Ablation of V1 INs lengthened the step cycle and slowed motoneuron burst frequency [18], while ablation of both V1 and V2b affects flexor-extensor alternation [19]. Furthermore, *in vitro* studies reconstructing SpIN circuits have shown that changing the ratio of excitatory and inhibitory neurons such as by adding more inhibitory V1 INs or excitatory V3 can influence rhythmic activity [17]. Therefore, changes to the SpIN network located in the intermediate gray matter, such as from a spinal cord injury, can affect motor output in an unpredictable manner.

In this study, we examined the combined roles of SpINs and propriospinal INs in laminae V-VIII in spinal levels L2-L4 in locomotion and whether intrinsic recovery after damage to this region is possible. By modifying a spinal excitotoxic kainic acid (KA) lesion model [8], we were able to lesion laminae V-VIII in spinal levels L2-L4, damaging both excitatory and inhibitory [20, 21] local and propriospinal INs. We observed detailed acute locomotor deficits that were not recovered over a three-month period. We developed a novel Random Forest classification model to combine all behavioral assessments and segregate KA-lesioned from uninjured animals, allowing us to further compare performance and recovery over time. Machine-learning based neuronal quantification indicates that neuronal loss in spinal levels L2-L4 in laminae V-VIII is critical and necessary for coordinated locomotion and cannot be compensated for by natural plasticity. This model allows us to further explore the potential of neurorestorative therapies, hence providing an ideal model for such studies.

## Material and methods

### Experimental design

#### Animals

Rats were housed in accordance with the European Union Directive and institutional guidelines. A total of 42 female Fischer 344 rats (Janvier Labs, Saint-Berthevin Cedex, France) weighing 180-200g (10 weeks old) at the time of the surgery were used for these experiments. 12 rats were used for pilot experiments with lesions in vertebrae T12. Animals with lesions that went into spinal level L2 were excluded from behavioral analysis (3 rats). Therefore, the total number was 9 rats, 4 controls and 5 KA animals. 30 rats were used vertebrae T13 injections. Of these, 21 rats were used in the first two-week experiment. One rat died after surgery and two control and 4 KA animals were excluded from the experiment as their lesion length did not fit the set criteria. Therefore, for the two-week experiment, a total of 14 rats were used, n = 7/group. For the three-month long-term experiment, a total of 9 rats were used. Two control animals and 1 KA animal were excluded for the same reason as mentioned above, resulting in a total of 6 rats, n = 3/group. An overview of the three experiments can be found in S1 Table. Rats were maintained in a 12 hour light-dark cycle at 22 degrees Celsius. Rats were group-housed in Type IV cages, with a maximum of 6 rats per cage. Animals had ad libitum access to water and food throughout the experiment. All experiments were planned and conducted according to the PREPARE and ARRIVE guidelines [22, 23]. Animals were split into control and KA groups prior to surgery by an unblinded colleague based on their baseline von Frey 1.4g filament performance.

#### Surgery

Experiments were conducted in accordance with the European Union Directive (Directive 2010/63/EU amended by Regulation (EU) 2019/1010) and approved by the local governing body (Regierungspräsidium Karlsruhe, Abteilung 3—Landwirtschaft, Ländlicher Raum, Veterinär- und Lebensmittelwesen, Germany (approval number: G-15/20). Female Fischer 344 rats were injected intramuscularly with a cocktail containing ketamine (62.6 mg/kg), xylazine (3.125 mg/kg), and acepromazine (0.625 mg/kg). After tail, paw and eye reflexes subsided, vertebra T12 or T13 was identified using the vertebra T10 landmark. For the pilot study, 12 animals received a laminectomy on vertebra T12 and received either one or two sets of bilateral 1μl, 1mM kainic acid (1mM; Tocris) injections. These injections were placed either rostral or rostral-mid vertebra T12. For the main experiment, 30 other animals received a laminectomy at vertebra T13 spanning 6mm. Following, three sets of bilateral 0.5μl injections of 1mM kainic acid were applied 0.4mm deep from the surface of the dura, 0.5mm laterally from the midline using a PicoSpritzer II (General Valve, Fairfield, NJ, USA) and a pulled glass capillary. Each set of injections was 3mm apart along the rostro-caudal axis. Control animals received saline injections at identical sites. Following, the overlaying muscles were sutured and the skin was stapled. Animals were transferred to a warm heating pad until all reflexes had returned. All surgeries were performed by a blinded experimenter. Two days postoperatively, rats were given buprenorphine (analgesic) (0.03mg/kg; Reckitt Benckiser) and ampicillin (167 mg/kg; Ratiopharm) subcutaneously three times daily. Bladder function was also manually assessed twice per day during this time however rats did not display bladder dysfunction.

#### Behavioral testing

Behavioral testing was performed throughout the experiment to assess sensorimotor deficits or recovery. Only animals with weight support on both hindlimbs (BBB score ≥ 9) were tested on the horizontal ladder, inclined beam, von Frey, Hargreave’s and CatWalk. All behavior tests were performed by blinded examiners and were performed during the same time of day, in the morning, to maintain consistency. An overview of the behavioral tests and their function can be found in S2 Table. A summary of the behavioral timelines can be found in S1 and S2 Figs.

#### Basso, Beattie, Bresnahan (BBB) score and subscore

For the two-week experiment, animals were tested 1, 3, 14 days after injury; for the three-month experiment, animals were tested 1, 3, 14, 30, 60 and 90 days after injury. Animals were placed into an open field where two blinded experimenters assessed gross hindlimb function [24]. A subscore with a total of 13 points looked at hindlimb function dependent on coordination (including toe clearance, paw position, trunk stability and whether the tail was up or down).

#### Even and uneven horizontal ladder

The horizontal ladder consisted of a custom, 1 meter setup with plexiglass and metal rungs. Rats were allowed to cross an elevated, unevenly spaced horizontal ladder (rungs spaced 2-3cm apart) five times to their home cage. After an hour break, animals were then allowed to cross an evenly spaced, elevated horizontal (rungs 3cm apart) ladder five times. Prior to baseline testing, animals were habituated on the evenly spaced ladder by allowing them to cross five times. Animals were not food deprived and no food reward was given at the end of the task. Following, animals in the two-week experiment were tested at baseline and 2 weeks after SCI. Animals in the three-month experiment were tested at baseline, 2, 4, 8, 12 weeks after injury. A Canon Legria HFR806 camera was placed perpendicular to the ladder set up and used to record performance. Animal fore- and hindlimb placement on the rungs were evaluated in slow motion using 25 frames per second and scored based on a previously established protocol [25]. A new uneven rung pattern was tested at each time point to prevent a learning effect. Percent hindlimb slips and overall hindlimb performance scores were calculated and averaged.

#### Inclined beam

Rats in the two-week experiment were tested on the inclined beam at baseline and 2 weeks after SCI. Rats in the three-month experiment were tested additionally 4, 8, and 12 weeks after injury. For this test, rats were placed onto an elevated and angled (10 degrees) 1.9cm narrow, wooden, circular 1 meter rod and trained to cross to reach their home cage. A green sheet was placed 0.3 meters below to gently catch rats who fell. Prior to baseline testing, animals were habituated by allowing them to cross five times or until they could cross without hesitation. For testing, rats were given the opportunity to cross the beam three times. A Canon Legria HFR806 camera was placed posterior to the beam and used to record inclined beam performance. Ability to complete the full task unassisted, time to cross and hindlimb slips were evaluated and scored. Each hindlimb step was given a maximum of 5 points, -1 point if foot placement was unstable; -2 points if the foot slipped off the beam but continued walking without stopping; -3 points the foot slipped off the beam and walking was interrupted; -4 points if the rat fell but was able to pull itself back up and complete the task; -5 points if the animal fell and did not complete the task. A time cut-off of 75 seconds was set. The total number of points was summed and normalized by the total possible number of points (total number of footsteps x 5). Scores for both hindlimbs over the three trials were averaged to calculate the average performance. Animals that did not complete the task received a 0.

#### Von Frey filament mechanical sensitivity testing

Dermatomes for the rat hindpaw are represented in L4 and L5 [26], regions in which part of the KA lesion is located, allowing us to evaluate changes in sensitivity. During habituation, animals were placed in a small box on a metal grid (Ugo Basile) for one hour for three consecutive days. Following, mechanical sensitivity was tested at baseline, 1 and 2 weeks after injury in the two-week experiment. Animals in the three-month experiment were tested additionally at 4, 8, 12 weeks after injury. Von Frey filaments 1.4g, 4g, 8g, 16g, 60g were used to cover the spectrum of light touch to nociceptive stimuli. Positive responses on each hindpaw were recorded after five applications. There were 4 minutes between each consecutive stimulation to prevent desensitization. Hindpaw responses on both sides were averaged. Von Frey filament testing was only performed with animals that had weight support and full plantar steps for both hindlimbs.

#### Hargreave’s thermal testing

During habituation, animals were placed in a small box on a plastic floor (Ugo Basile) for one hour for three consecutive days. Following, baseline testing was performed in which a laser machine (50 units, maximum 30 seconds) was placed beneath each hindpaw and response time was recorded. Each hindpaw was tested four times and there were 8 minutes between each stimulation to prevent desensitization and damage. If there was urine or feces in the box, two minutes passed post-removal before testing resumed. This test was performed at baseline, 1, 2 weeks post-injury for animals in the two-week experiment. Animals in the three-month experiment were tested additionally 4, 8, 12 weeks post-injury.

#### CatWalk gait analysis

All animals were transported to the Interdisciplinary Neurobehavioral Core (INBC) in Heidelberg three days prior to testing for acclimatization. Following, a CatWalk Noldus XT was used to perform the CatWalk experiments. The following settings were used: camera height (48 cm); walkway width (7cm); green intensity threshold (0.1); camera gain dB (18.10); red LED ceiling light v (17.7); green LED light (16.0).

For this test, animals were trained to traverse the walkway without stopping. Habituation was performed one day prior to testing. On the day of the experiment, the first 5 runs where the maximum speed variation was under 50 and had speed variation less than ten were taken. All analysis was performed using the CatWalk Noldus XT software. The pLDA score was calculated according to the previously established protocol [27]. Animals without weight support were not included in the pLDA analysis. Following gait analysis, animals were transported back to the ZOUP Animal Facility Heidelberg for completion of the experiment. CatWalk gait analysis was performed once at the end of the two-week and three-month experiments, one day prior to sacrificing.

#### Classification of animal behavioral performance

We were also interested in a correct separation of KA-injured and non-injured animals without post-hoc assessment of the lesion-length. For this, extracted features were obtained by the performed behavioral tests (scores) and trained classification models using Random Forests which uses an ensemble of decision trees and aggregates their votes. Two independent approaches were explored: for the first approach (FULL), behavioral scores from all behavioral tests performed with the animals were extracted, namely (i) BBB, (ii) Horizontal Ladder, (iii) Inclined Beam, (iv) Sensory and the (v) CatWalk tests. For the second approach (ECO), the feature set was deliberately reduced to the BBB and inclined beam tests in order to provide a prediction model which relies only on a few fast and economically convenient behavioral tests.

Since the number of repetitions (trials) for most behavioral tests at each time point was different (1 to 5 repetitions), only 3 individual observations were generated per animal: For the Catwalk, horizontal ladder, inclined beam and all sensory tests from the first three trials were taken, for the BBB Scores the same score was used for each observation. In order to guarantee uniformity of the data, animals KA#5 and KA#7 were excluded from both classification approaches, since they did not perform the CatWalk test. In addition, all animals which were not able to successfully master the inclined beam test received 75s (stop criterion) for the time parameter, and 50 steps for the steps parameter. For each observation, 28 features were extracted: 2 BBB, 4 horizontal ladder, 4 inclined beam, 3 sensory and 15 CatWalk parameters. A detailed listing can be found in S3 and S4 Tables.

In this way, 21 observations were generated for the control group (3 x 7 control animals) and 15 for the KA group (3 x 5 KA animals), with a combined total of 36 observations (each observation included 28 behavioral parameters for the FULL model, and 6 behavioral parameters for ECO model).

Using a 10x 5 -fold cross-validation technique to avoid overfitting to the data, all available trials were divided into test and training data. A Random Forest classification model was trained to segregate observations between the control and KA group. One thousand trees were chosen for the decision aggregation and the square root of the number of features was chosen as the splitting criterion as previously published [28]. The mean of all aggregated votes and the accuracy obtained over all cross-validated results were reported. Chance was calculated using a Wald interval (aWI) for with a p value of 0.05 [29].

In addition, behavioral scores which contained the most discriminative information between KA-injured and non-injured animals were investigated. Using a Random Forest model, this feature ranking can be obtained by assessing the Gini Index for a feature at each split. The mean decrease of the Gini Index in magnitude was calculated for each feature over all cross-validated results.

Lastly, the votes obtained via decision aggregation per observation was assessed and correlated to the actual lesion length using Spearman’s correlation analysis.

### Tissue processing and immunohistochemistry

#### Transcardial perfusion

All animals were euthanized with an overdose of anesthesia cocktail (ketamine, xylazine, and acepromazine) and transcardially perfused with saline and fixed with 4% paraformaldehyde (PFA). All cords were dehydrated in 30% sucrose for one week. Spinal cords were cryosectioned into 25 μm thick coronal sections in a 1:7 series on super frosted, charged glass slides. All tissue was stored at -20°C until use.

#### Immunohistochemistry

Slides were first dried for 15 minutes at room temperature. Slides were washed 3x with tris-buffered saline (TBS) and sections were permeabilized with a blocking solution of 5% donkey serum (Equitech-Bio Inc), 0.25% Tx-100 (neoLab Migge GmbH) in TBS for two hours at room temperature. Primary antibody (Merck guinea pig anti-NeuN 1:1000, Cat# ABN90) was added in 0.1% TX-100 and 1% donkey serum and incubated overnight at 4°C. The next day, slices were washed three times with 1% serum in TBS and incubated in secondary antibody (Dianova Alexa-Fluor 594 anti-guinea pig 1:300, Cat# 706295148) with DAPI (1:2000) for four hours at room temperature. Slides were washed again in TBS before being mounted on a cover-slip with Fluoromount G (Biozol, Cat # SBA-0100-01).

### Histological analysis

#### Lesion length and exclusion criteria

To determine the lesion length in the rostro-caudal axis, neuronal loss was identified and quantified in coronal sections immunostained for NeuN using an Olympus BX53 microscope. The number of slices with neuronal loss in a complete series were quantified and then multiplied by 25 microns x 7 to determine the length of the lesion. Within this region, the three slices with the greatest neuronal damage (ie the three bilateral injection sites) were identified as the lesion epicenters. All histological analysis was performed by a blinded experimenter. To determine if animals were appropriately lesioned and to ensure that the observed behavior is due to the desired lesion, we applied specific exclusion criteria based on the histological results. If control animals had a lesion length greater than 2800μm in the rostro-caudal extent, they were excluded from the experiment. If KA animals had a lesion length smaller than 6000μm, they were excluded from the experiment as they did not show consistent behavioral deficits.

#### NeuN quantification

To quantify NeuN-positive cells in laminae I-IV, V-VIII and IX in an unbiased fashion, the following workflow was developed (S3 Fig). Spinal cord sections were imaged using the Olympus XT1000 confocal microscope. Z-Stacks of 1.5 micron step size were acquired of each epicenter with the 10x magnification objective (UPlanSApo, 10x/0.40, infinity/0.17/FN26.5). Tiles were stitched in ImageJ/Fiji using the Grid/Collection stitching plugin with the following settings: Unknown Position; linear blending fusion method; 0.30 regression threshold; 2.5 max/avg displacement threshold; 3.5 absolute displacement threshold; and with subpixel accuracy [30]. One coronal section (25μm) was analyzed per bilateral injection site (lesion epicenter). A total of three injection epicenters per spinal cord were analyzed.

To determine the correct spinal level, the maximum intensity projection (MIP) of the Z-stack of each injection epicenter was computed and registered with the matching spinal cord atlas section [31]. A customized ImageJ/Fiji script was developed to semi-automate the registration process (https://github.com/cberri/2D_Registration_BrainAtlas_ImageJ-Fiji). The ImageJ/Fiji script uses the BUnwarpJ plugin (https://imagej.net/plugins/bunwarpj/) to register the MIP images with the corresponding selected atlas map (T13-L4). Indeed, the atlas section that best correspond to the spinal cord gray matter was selected and registered to the appropriate spinal section using land markers. The laminae I-IV, V-VIII and IX regions of interest (ROIs) were cropped with ImageJ/Fiji polygon selection tool and saved [32]; manual verification was performed. One coronal section (25μm) was analyzed per bilateral injection site (lesion epicenter). A total of three epicenters (6 ROIs) per spinal cord were analyzed.

To enhance signal to noise ratio and normalize the background across images, all the individual Z-stack tiles were processed using the ilastik pixel classification workflow [33]. Two label classes (foreground NeuN and background) were used to differentiate NeuN-positive pixels from background pixels and the resulting foreground probability maps stitched in ImageJ/Fiji with the Grid/Collection stitching plugin. Ilastik pixel classification training was performed prior on 10 sample images. The 2D ROIs generated as described above were over-imposed on the 3D probability map and the pixels outside filled with zero values using the ImageJ/Fiji Clear Outside plugin.

3D automated image segmentation was performed on the processed ROI probability maps using cellpose *Nuclei* pretrained model in a customized Jupyter Notebook (cellpose version 0.6.2; [34]). The labeled images were imported in arivis Vision4D (arivis AG) for visualization. Minor mistakes in the segmentation were manually corrected using arivis Vision4D 3D magic wand tool. Segments smaller than 100μm^3^ were excluded. The NeuN counts were exported in an Excel table and normalized by the selected ROI image volume (counts divided by (ROI area x 25microns)). Normalized NeuN counts for the respective left and right sides were summed to get the total NeuN count per volume at a specific spinal level. Total NeuN in L2-L4 was calculated by summing the total NeuN in each individual spinal level. To determine if there were differences between groups, the total averages per group were compared.


$$Normilized\;NeuN\;Counts=\frac{Total\;NeuN\;Counts}{ROI\;Area*Z\;Height}$$

#### Workstation hardware specification

All image analysis was performed on a workstation equipped with a Nvidia RTX 2070 Super GPU, 128 GBs RAM and 10 Cores Intel i7 processor.

#### Software and data accessibility

All custom code written for this project can be found under the following link: https://github.com/naemikuehn/lumbarcordanalysis.git.

Study data were deposited at the Open Data Commons for Spinal Cord Injury (ODC-SCI; RRID:SCR_016673). The ODC-SCI is a secure, cloud-based repository platform designed to share research data [35] (DOI: 10.34945/F5DK52, https://odc-sci.org/data/938).

#### Motoneuron analysis

To determine the number of surviving motoneurons at each lesion epicenter, NeuN-positive neuronal cell bodies in lamina IX were quantified using a similar method as previously published [36]. The correct lamina was determined using the Spinal Cord Atlas [31]. All NeuN-positive cells with a cell body larger than 919.632μm^2^ in laminae IX were counted as motoneurons and then analyzed in Graphpad Prism 6 to determine if there was a correlation between remaining motoneurons and behavioral performance. One coronal section was analyzed per bilateral injection site (lesion epicenter). A total of three epicenters per spinal cord were analyzed. All analysis was performed by a blinded investigator.

White matter area analysis

To determine if white matter was damaged, the area of white matter of the spinal cord cross-sectional area at each given epicenter was calculated. Briefly, slides were subjected to an Eriochrome Cyanine staining and imaged using a XC30 camera and an Olympus Bx53 microscope (Olympus, Hamburg, Germany). White matter area was calculated in Image J. One coronal section was analyzed per bilateral injection site (lesion epicenter) and a total of three epicenters per spinal cord were analyzed. White matter area of the spinal cord was averaged per animal and compared between the control and KA groups. All analysis was performed by a blinded investigator.

#### Heatmap analysis

Behavioral tests and neuronal quantification at spinal levels L2-L4 were compared between individual KA-lesioned animals. All values were normalized to the highest control value and reported as a percentage except for lesion length. For lesion length, all values were normalized to the largest lesion extent, 15,925μm for the short-term experiment and normalized to 8,575μm for the long-term experiment.

#### Statistical analysis

All data is shown as mean ± the standard error of the mean unless otherwise reported. A repeated measures 2-way ANOVA with Sidak’s post hoc multiple comparisons test was calculated to evaluate differences between the two groups in tests that were repeatedly performed over a period of time (BBB score, BBB subscore). Unless otherwise noted, all reported p-values are group differences p-values. All other tests underwent Shaprio-Wilk’s normality test. If normally distributed, a Welch’s unpaired t-test to determine differences between the two groups, if not normally distributed, a Mann-Whitney test was used (inclined beam, horizontal ladder, von Frey Filament testing, Hargreave’s, CatWalk, motoneuron, white matter area, NeuN counts). Linear regression analysis was performed to compare lesion length, motoneuron counts and white matter sparing with behavior. Shapiro-Wilk’s normality test was performed prior to Pearson’s (normally distributed) or Spearman’s (not normally distributed) correlation analysis to analyze the relationship between the two variables. All statistical tests were performed with Prism 6 and Prism 9 software (Graphpad, San Diego, CA, USA).

#### Schematics

Schematics were created with BioRender.com, Microsoft Powerpoint and Graphpad Prism.

## Results

### Targeting intermediate gray matter for neuronal loss in lumbar spinal levels L2-L4 with three sets of bilateral KA injections

**Fig 1:**
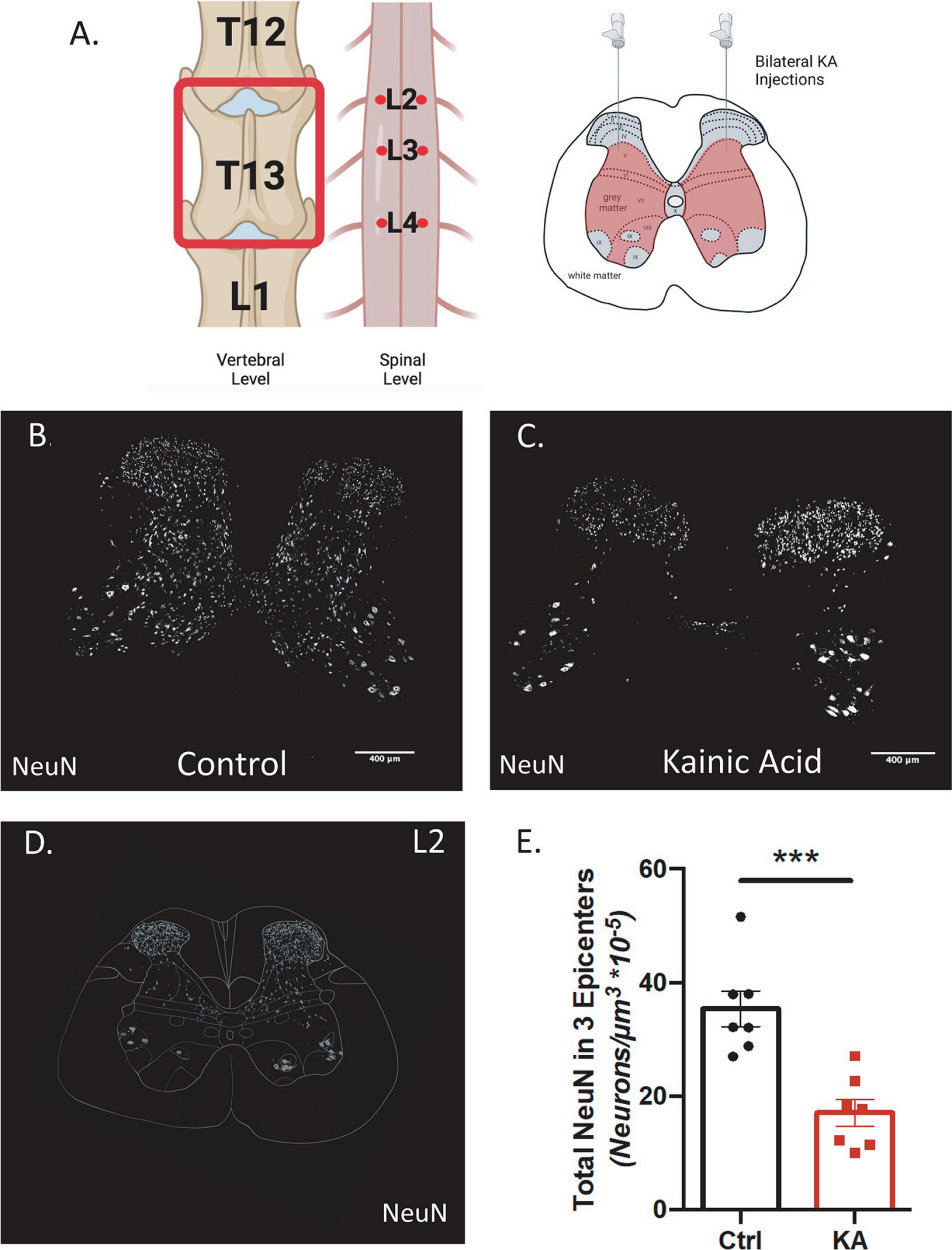
Defined kainic acid (KA) injection parameters creates selective intermediate gray matter damage in the lumbar spinal cord. (A) Schematic shows vertebral and spinal injection levels in the rostral and caudal axis as well as in the dorsal-ventral axis. (B-C) Representative images of lesion epicenters stained with NeuN to visualize neuronal loss in the control and kainic acid spinal cord. (D) Representative image of atlas overlay on coronal spinal cord section at spinal L2. E, Total NeuN in laminae V-VIII at lesion epicenters were quantified and compared (Welch’s unpaired t-test, *** p = 0.0007, n = 7 animals per group).

In order to create a lesion that targeted various SpINs contributing to locomotion, KA injection parameters were refined to create selective intermediate gray matter damage in the lumbar cord with a behavioral readout. This was achieved with three bilateral KA injections performed at vertebral level T13 in female Fischer rats, each consisting of 0.5μl of 1mM KA injected 0.5mm from the midline at a depth of 0.4mm (Fig 1A–1C). All gray matter neurons were visualized with NeuN with a custom workflow to confirm damage in the target region. The ImageJ/FIJI plugin “registration of pairs” was used to align the spinal atlas over 3D confocal coronal section Z-stacks for determination of the spinal level and target laminae (Fig 1D). To quantify the number of NeuN-positive neurons in laminae V-VIII from spinal levels L2-L4 in a consistent and unbiased fashion, the newly developed combined image analysis workflow was used: ilastik pixel classification, 3D segmentation using cellpose and arivis Vision4D for visualization and segmentation correction (S3 Fig). Kainate lesions significantly reduced the number of NeuN-positive neurons in laminae V-VIII to 17.07 ± 2.38 neurons/μm^3^*10^−5^ in comparison to 35.35 ± 3.13 neurons/μm^3^*10^−5^ in the control group (Fig 1E). With this experiment, we have confirmed that injecting KA with these defined parameters creates intermediate gray matter damage in the spinal levels L2-L4 of the spinal cord.

### KA rats show deficits in gross hindlimb function, rhythmic and skilled walking, coordination, balance and gait after two weeks

**Fig 2:**
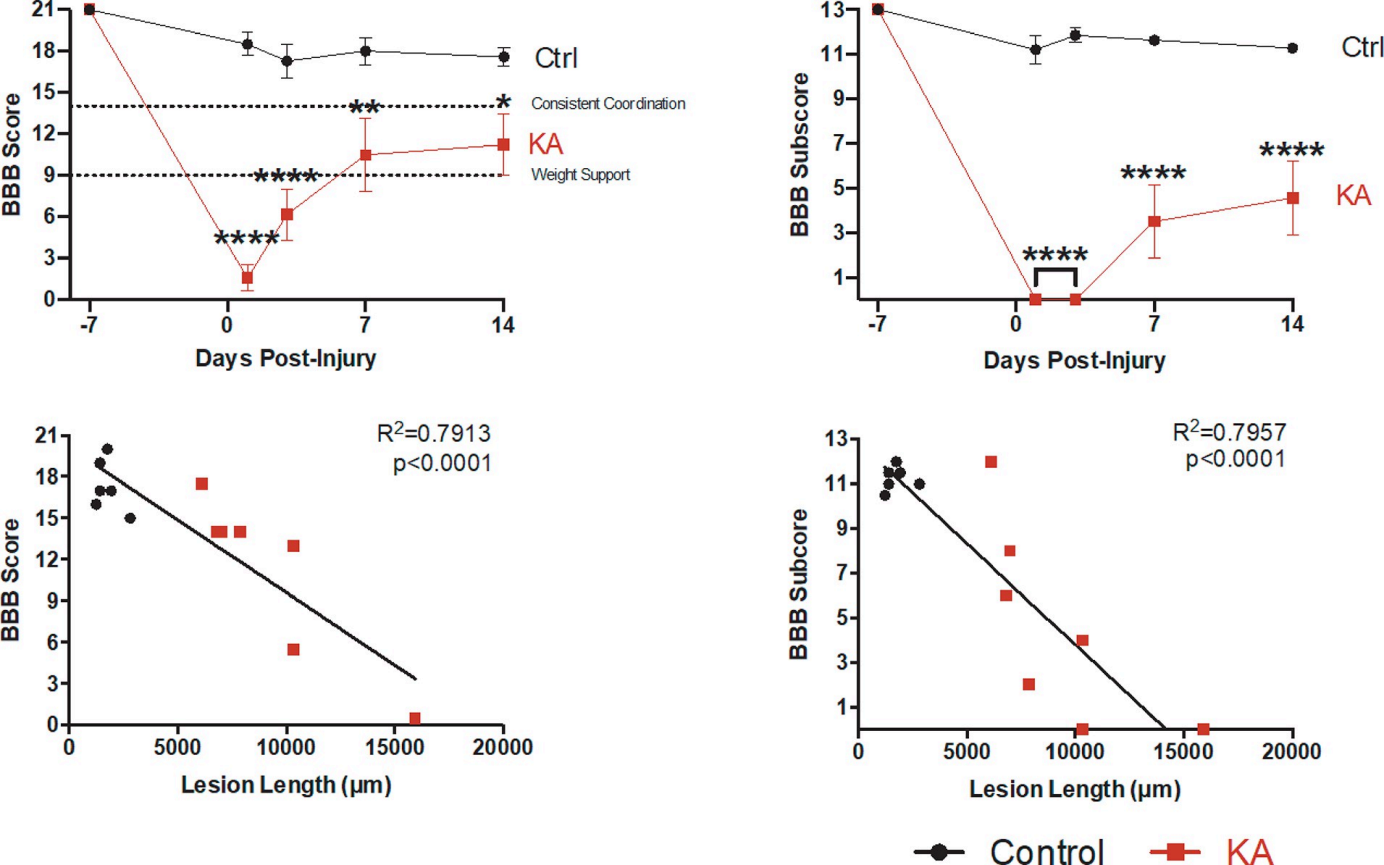
KA-injected rats display deficits in gross hindlimb function after two weeks. A score below 9 indicates that animals do not have full weight-supported steps but a score above 14 indicates consistent coordination while walking. (A-B) KA-injected animals have a significantly lower BBB score and sub-score after two weeks in comparison to the control (BBB score: 2-way ANOVA with Sidak’s post hoc test, group p < 0.0001; BBB sub-score: 2-way ANOVA with Sidak’s post hoc test, group p < 0.0001). (C-D) There were significant correlations between lesion length in the rostro-caudal axis and BBB score and sub-score (Linear Regression Analysis, BBB score p < 0.0001, R^2^ = 0.7913; BBB sub-score p < 0.0001, R^2^ = 0.7957). For all data, n = 7 animals per group; * p ≤ 0.05, ** p ≤ 0.01, **** p ≤ 0.0001.

To assess the behavioral deficits induced by a discrete KA gray matter SCI, we performed a series of behavioral tests two weeks post-injury. The Basso-Beattie-Bresnahan (BBB) was performed 1, 3, 7 and 14 days post-injury (dpi) to evaluate gross hindlimb motor function (Fig 2A and 2B). We observed animals with a KA lesion have significant deficits in gross hindlimb function 1 day after injury which stabilizes one-week post-SCI (Fig 2A: Mean BBB scores after 14 days: control = 17.57 ± 0.69, KA = 11.21 ± 2.25) as well significant deficits in hindlimb function dependent on coordination (Fig 2B: mean BBB subscore control = 11.29 ± 0.18, KA = 4.57 ± 1.67). These deficits correlate to lesion length in the rostro-caudal axis (Fig 2C and 2D: BBB score r = -0.8254 and p = 0.0004, BBB subscore r = -0.7138 and p = 0.0041, Spearman’s correlation). Two animals did not have weight support. The animal with the greatest lesion length had the lowest BBB score, however the second animal without weight support had the same lesion length as one that did, indicating that lesion length is not the only determining factor for behavioral performance.

**Fig 3:**
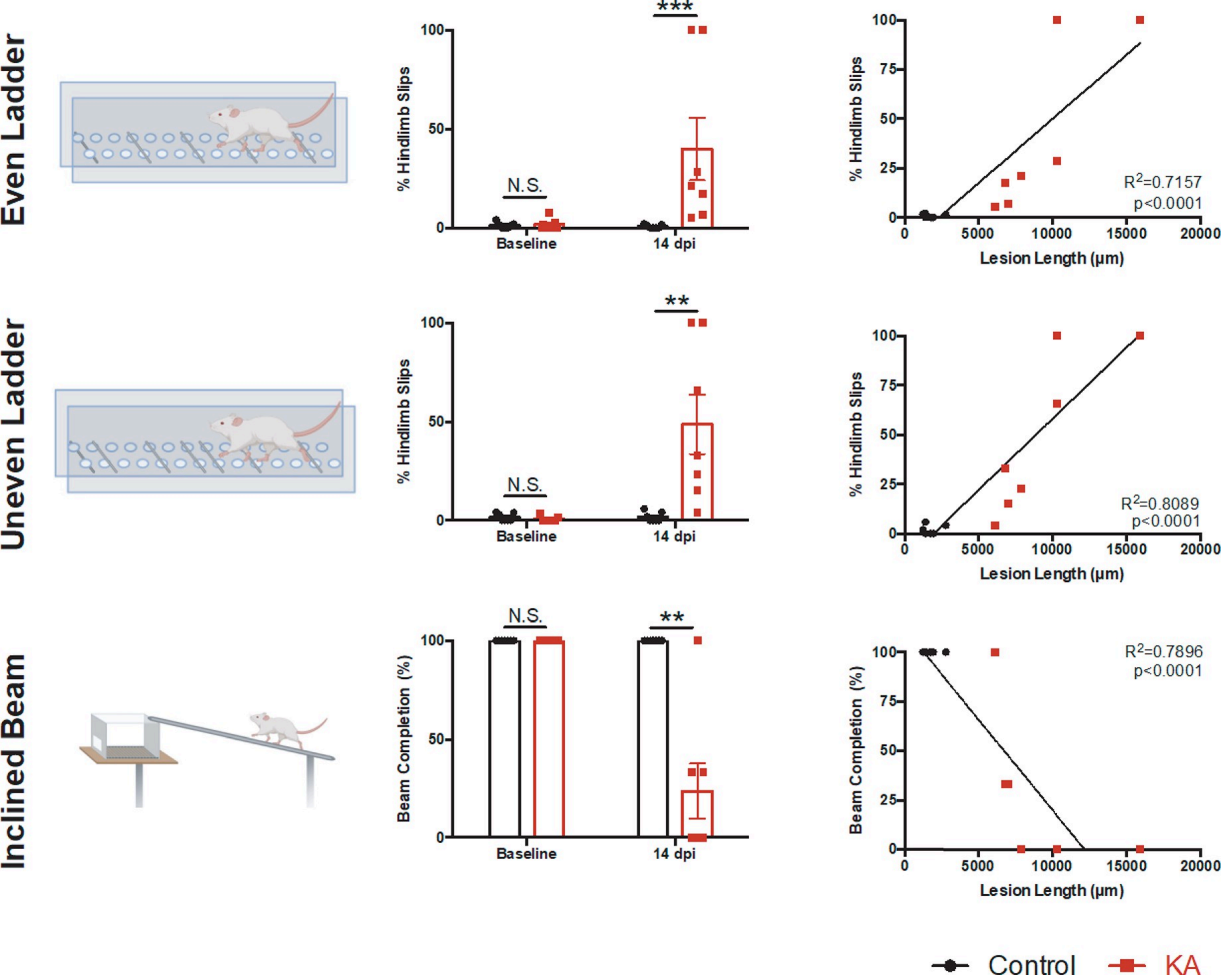
KA-injected rats display deficits in rhythmic and skilled-walking, coordination and balance, correlating to lesion length. (A) Schematic of the evenly-spaced rungs of the horizontal ladder. (B) Percent hindlimb slips were compared two weeks post-KA SCI (Mann-Whitney test, at baseline p = 0.9184, at 14 dpi p = 0.0006). (C) Correlation analysis is significant between hindlimb slips and lesion length in the rostro-caudal axis (Linear Regression Analysis, p < 0.0001, R^2^ = 0.7157). (D) Schematic of the unevenly spaced rungs of the horizontal ladder. (E) Percent hindlimb slips were compared two weeks post-KA SCI (Mann-Whitney test, at baseline p = 0.4126, at 14 dpi p = 0.0023). (F) Correlation analysis is significant between hindlimb slips and lesion length in the rostro-caudal axis (Linear Regression Analysis, p < 0.0001, R^2^ = 0.8089). (G) Schematic of the inclined beam behavioral test. (H) Ability to complete the inclined beam task was significantly impaired in KA SCI rats (Mann-Whitney test, at 14dpi p = 0.0047) (I) Correlation analysis is significant between ability to complete the inclined beam and lesion length in the rostro-caudal axis (Linear Regression Analysis, p < 0.0001, R^2^ = 0.7896). For all data, n = 7 animals per group; N.S. stands for not significant, ** p ≤ 0.01, *** p ≤ 0.001.

Although the BBB score is useful in characterizing SCI animals, it is not as sensitive for tasks we believe are affected by our lesion model. Therefore, we opted for tasks specific for our lesion paradigm such as rhythmic and skilled walking, where animals were tested on an even horizontal ladder, uneven horizontal ladder [37] and inclined beam [38] (Fig 3). KA rats show significantly higher number of hindlimb slips on both the even and uneven horizontal ladders indicating deficits in rhythmic walking (Fig 3B: even ladder controls = 0.70 ± 0.34%, KA = 39.91 ± 15.81%) and coordination (Fig 3E: uneven ladder controls = 1.71 ± 0.91%, KA = 48.68 ± 15.12%). Only 3 of the 7 KA rats were able to complete the inclined beam test (Fig 3H: controls = 100 ± 0% completion, KA = 23.81 ± 14.02%). Those KA rats which did complete the beam had a lower performance score (Controls = 95.66 ± 1.25%, KA = 21.19 ± 13.32%) further indicating deficiencies in coordination and balance (S1 Video). These behavioral tests significantly correlated to lesion length in the rostro-caudal axis (Fig 3C, 3F, 3I: even horizontal ladder r = 0.8490 and p = 0.0003, uneven horizontal ladder r = 0.8401 and p = 0.0004, inclined beam r = -0.8944 and p < 0.0001, Spearman’s correlation).

**Fig 4:**
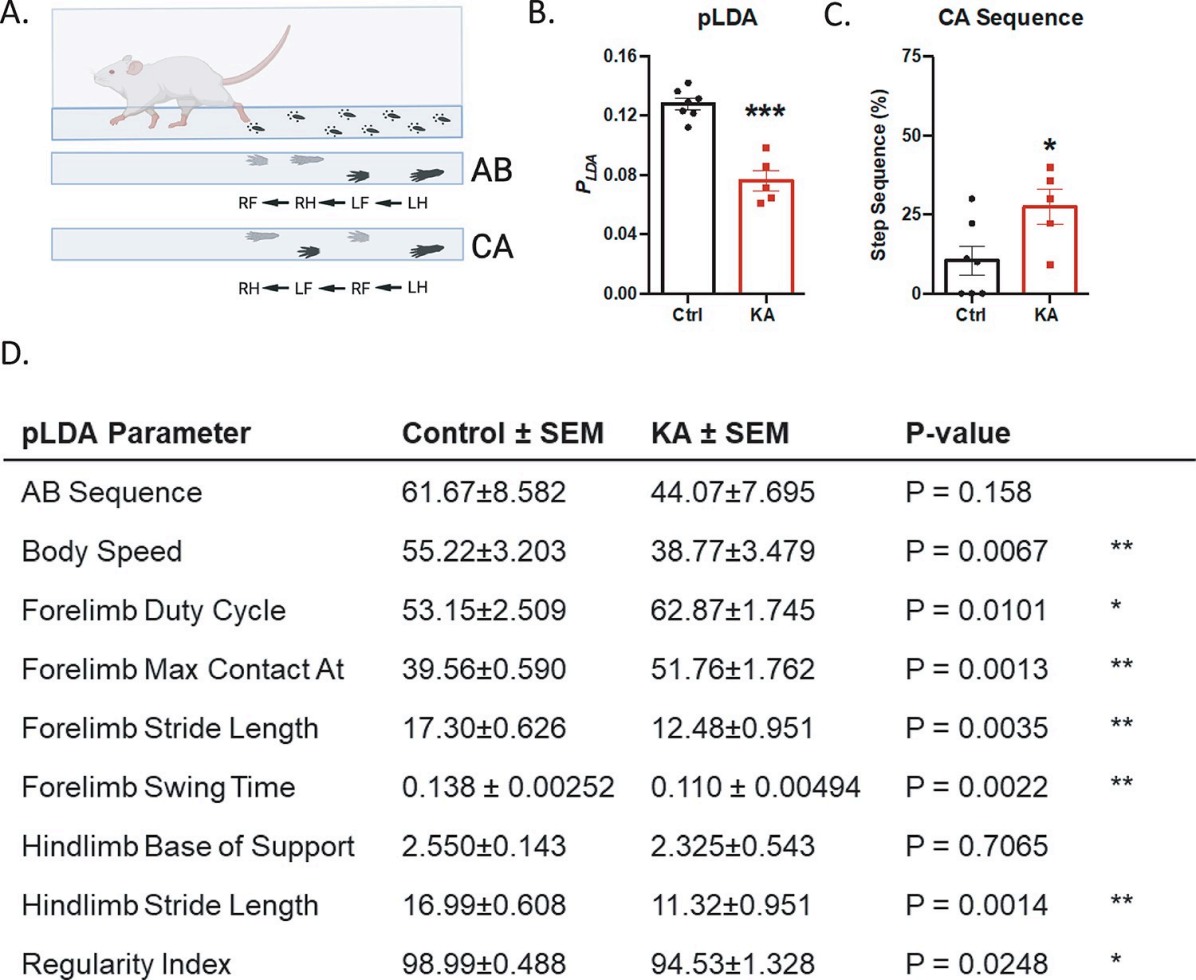
CatWalk gait analysis highlights deficits in SCI-related gait parameters two weeks post-injury. (A) Schematic of the CatWalk behavioral test with AB (alternate) vs CA (cruciate) step sequences. The AB step sequence requires a shift from hind-to forelimb (or fore-to hindlimb) on the ipsilateral side of the body while the CA step sequence requires a shift from fore-to forelimb (or hind-to hindlimb) on the contralateral side. (B) Comparison of the pLDA gait score (parameter linear discriminant analysis) shows significant differences (Welch’s unpaired t-test, p = 0.0006). (C) Comparison of CA sequence shows significant differences (Welch’s unpaired t-test, p = 0.0415). (D) Analysis of individual pLDA CatWalk parameters. Significance determined using Welch’s unpaired t-test. For all data n = 7 control and n = 5 KA animals; * p ≤ 0.05, ** p ≤ 0.01, *** p ≤ 0.001.

Static and dynamic parameters of each forelimb and hindlimb as well as the whole-body automated gait analysis was performed with the CatWalk (Noldus). Gait analysis also further investigates the role of propriospinal INs connecting the cervical and lumbar enlargements [39–42] An algorithm (parameter-combined linear discriminant analysis, pLDA) that combines 9 SCI-related gait parameters found to be predictive of injured vs uninjured thoracic SCI rat models was applied. In comparison to the controls, KA rats have a significantly lower pLDA score two weeks after SCI (Fig 4B: controls = 0.13 ± 0.01, KA = 0.076 ± 0.01). Separation into the nine individual parameters indicated significant differences in all except for hindlimb base of support and alternate B (AB) step sequence (Fig 4D). Additionally, we found KA-lesioned rats had a significantly higher cruciate A (CA) step sequence in comparison to the controls (Fig 4A and 4C: controls = 10.48 ± 4.50%, KA = 27.41 ± 5.46%). A closer analysis of the rhythmic components of gait showed significant differences in forelimb swing time, stand time and duty cycle (S4A, S4C, S4E Fig) However, the hindlimb parameters were not significantly different (S4B, S4D, S4F Fig).

**Fig 5:**
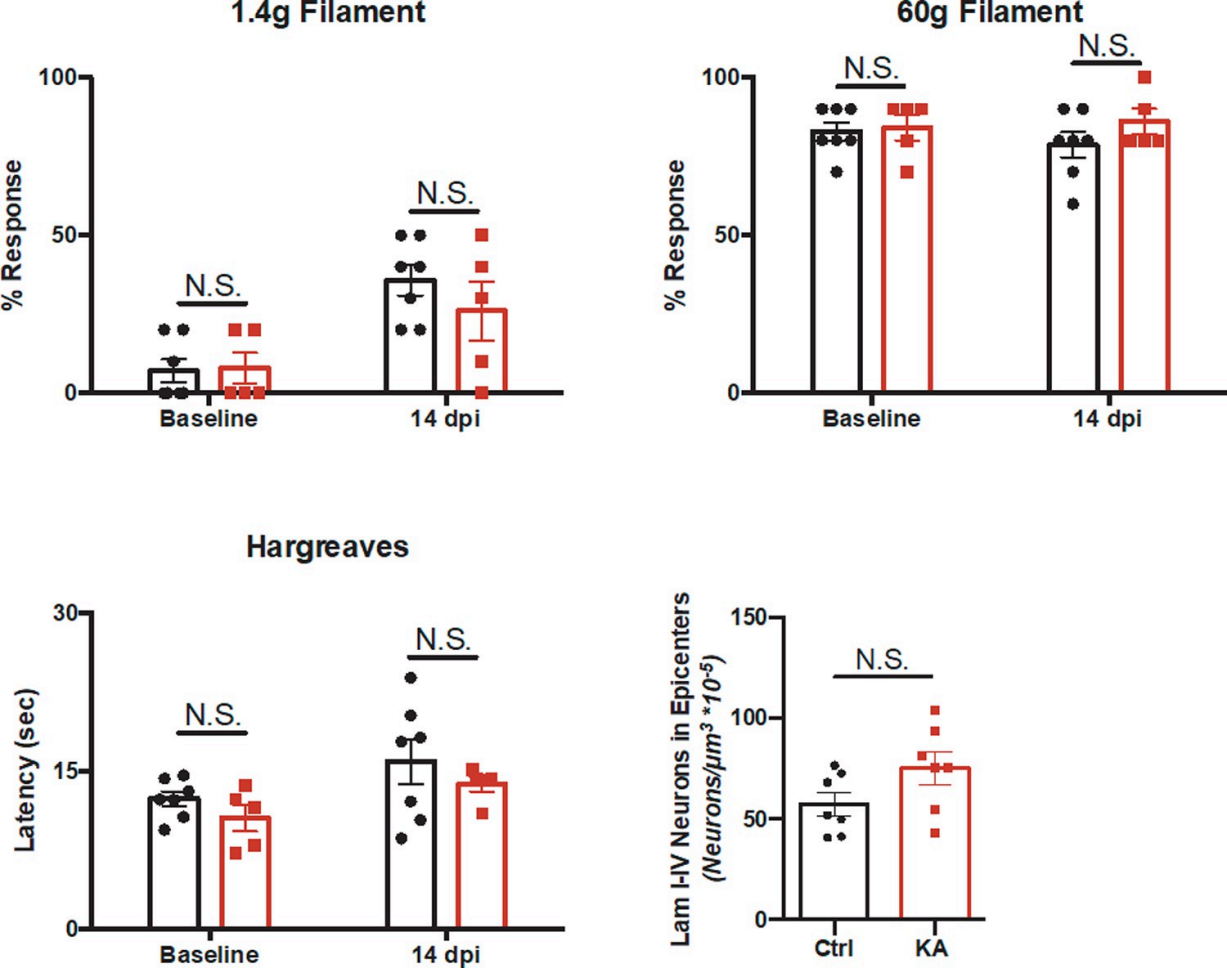
KA animals do not show differences in mechanical or thermal sensitivity after two weeks. (A-B) Hindpaw stimulation with 1.4g innocuous and 60g noxious von Frey hair filaments did not show significant differences at baseline or after two weeks (Mann-Whitney test, 1.4g at baseline p > 0.9999; Welch’s unpaired t-test, 1.4g at 14 dpi p = 0.3875; 60g at baseline p = 0.8222, 60g 14 dpi at p = 0.2217). (C) Hargreave’s hindpaw stimulation did not show differences in thermal sensitivity between the two groups (Welch’s unpaired t-test, at baseline p = 0.2496, at 14 dpi p = 0.3727). (D) Quantification of neurons in laminae I-IV did not show significant differences between the two groups (Welch’s unpaired t-test, p = 0.0926). For all data, n = 7 control and n = 5 KA animals. N.S. stands for not significant.

To further investigate the specificity of our lesion and ensure we did not disrupt the dorsal horn circuitry that may lead to sensory deficits [9, 43], we performed several sensory tests. We did not find any significant differences in mechanical and thermal sensitivity in animals two weeks post-KA SCI (Fig 5A–5C). Additionally, quantification of neurons in laminae I-IV did not show significant differences between the two groups (Fig 5D). These results suggest that KA did not damage the dorsal horn as there were no significant sensory differences after two weeks.

Together, this battery of behavioral assessments of rats with a lumbar intermediate gray matter SCI shows gross hindlimb deficits, impaired rhythmic walking, coordination, balance and gait but no differences in sensory function two weeks post-injury. Lesions in a mixed cohort which we found to be primarily in spinal levels T13/L1 and not overlapping into spinal L2 did not show significant gross hindlimb and coordination deficits at baseline and two weeks after injury. At baseline the BBB score of both the control and KA groups was 21 ± 0.0. At 14dpi, the BBB score of the control group was 20.63 ± 0.38 and the KA group was 18.20 ± 0.92 and not significantly different (p = 0.0642, unpaired Welch’s t-test, n = 4 control and n = 5 KA animals). The BBB subscore at baseline for both the control and KA groups was 13.00 ± 0.0. Two weeks after injury, the BBB subscore for the controls was 12.75 ± 0.25 and for the KA animals was 10.8 ± 0.80 and was also not significantly different (p = 0.0750; unpaired Welch’s t-test, n = 4 control and n = 5 KA animals). Finally, the percent hindlimb slips on the uneven ladder for the controls at baseline was 2.15 ± 1.44 and for the KA animals was 2.26 ± 1.20% (p = 0.9557, unpaired Welch’s t-test, n = 4 control and n = 5 KA animals). Two weeks post-lesioning, the percent hindlimb slips for the controls was 1.19 ± 0.79% and for the KA group was 4.29 ± 2.24% (p = 0.2470, unpaired Welch’s t-test, n = 4 controls and n = 5 KA animals). Together, this indicates that the observed deficits are specific to this lesion in spinal L2-L4.

### Neuronal quantification highlights role of spinal level L2-L4 INs in coordination and balance

**Fig 6:**
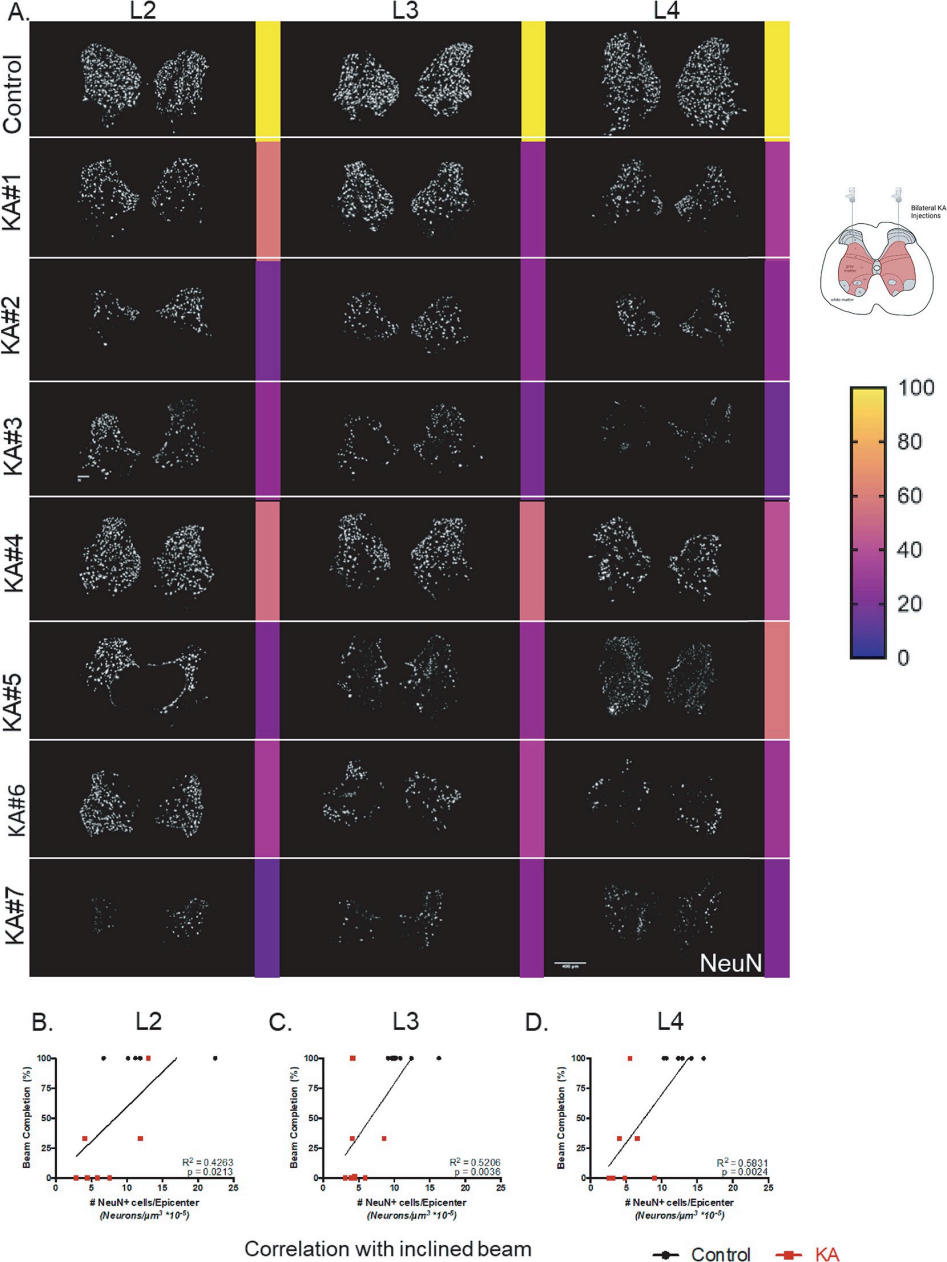
Laminae V-VIII neuronal loss in spinal levels L2-L4 correlates with deficits in balance and coordination deficits. (A) Visualization of NeuN in laminae V-VIII in spinal levels L2-L4 injection epicenters using ilastik MIPs. Single images of the KA animals in comparison to a representative control animal. Number of neurons are quantified and normalized to the average of the controls, shown as a percentage (0% in violet, 100% in orange, above 100% in yellow). (B-D) Correlation of NeuN in spinal level L2-L4 in comparison to inclined beam performance (Linear Regression Analysis, L2 inclined beam p = 0.0213, R^2^ = 0.4263; L3 inclined beam p = 0.0036, R^2^ = 0.5206; L4 inclined beam p = 0.0024, R^2^ = 0.5831; n = 5–7 animals per group).

We next wanted to determine whether neuronal loss at a specific spinal level significantly contributes to behavioral deficits. Using the above-mentioned NeuN quantification method in Fig 1, we further analyzed the number of NeuN-positive neurons in laminae V-VIII in spinal L2-L4 in KA animals and normalized this to the average controls. In comparison to the average controls, there is a reduction of NeuN in all KA injected spinal levels except for KA#1 in spinal level L2 (Fig 6A). We found that the number of NeuN-positive neurons in all three spinal levels L2-L4 significantly but moderately correlates to the inclined beam performance (Fig 6B–6D: L2 p = 0.0219 and r = 0.6718; L3 p = 0.0031 and r = 0.7561; L4 p = 0.0037 and r = 0.7756, Spearman’s correlation, respectively). Additionally, correlation analysis comparing combined total NeuN in all three levels to inclined beam performance was stronger than the separated levels (r = 0.8104, p = 0.0010, Spearman’s correlation) This data indicates that intermediate gray matter IN loss in each spinal level (L2-L4) correlates with coordination and balance performance (S5 Fig) and that together they are required for the overall effect.

### Lower motoneurons and white matter area at the L2-L4 lesion epicenters do not correlate with behavioral performance

**Fig 7:**
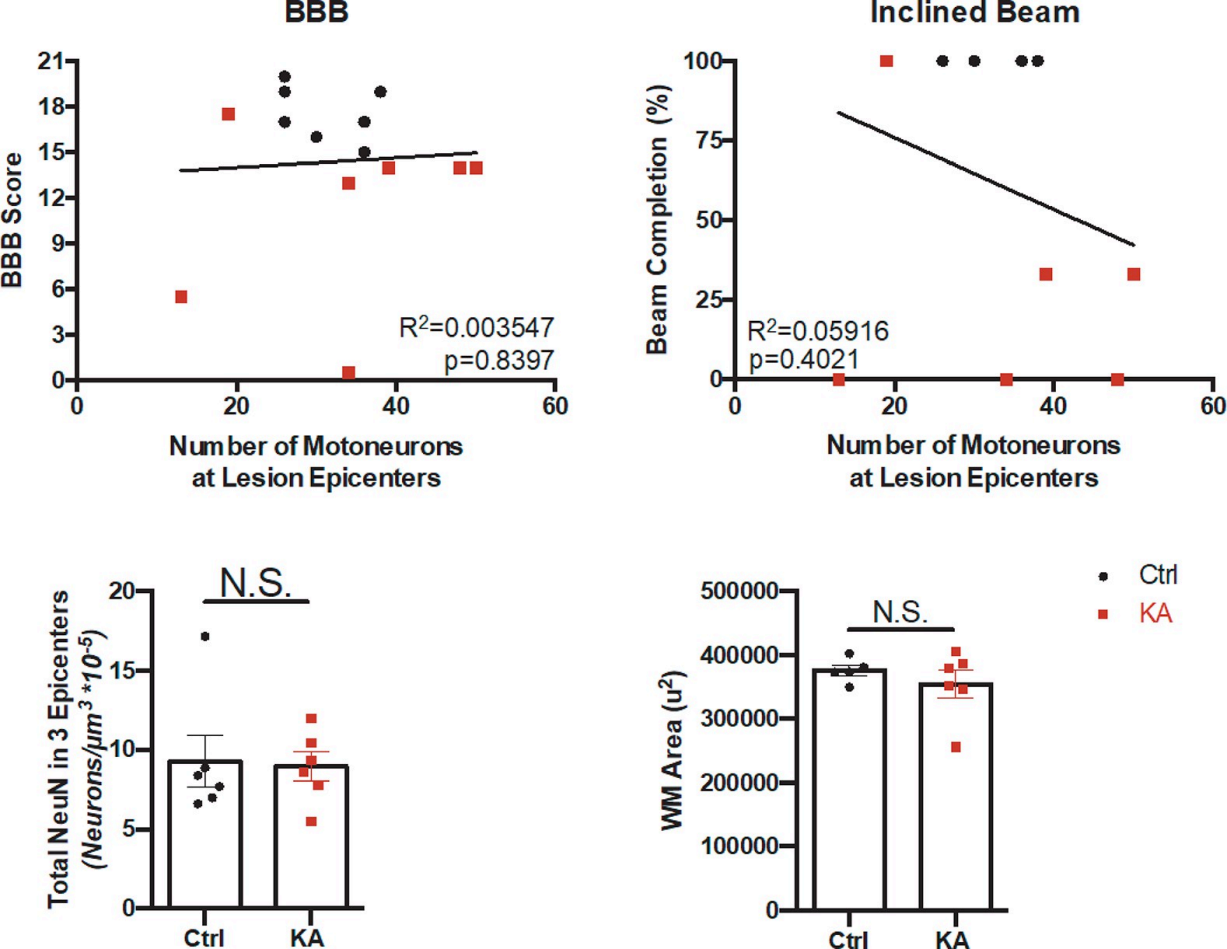
Motoneuron counts and white matter area at spinal L2-L4 lesion epicenters do not significantly affect behavioral deficits. (A-B) Sum of remaining motoneurons at lesion epicenters does not correlate with BBB score nor inclined beam completion (Linear Regression Analysis, BBB score p = 0.8397, R^2^ = 0.003547; Beam Completion p = 0.4021, R^2^ = 0.05916, n = 7 animals per group). (C) Quantification of total number of NeuN+ in lamina IX of the lesion epicenters does not show significant differences between the two groups (Welch’s unpaired t-test, p = 0.8611, n = 6 per group). (D) Average white matter area at the three lesion epicenters is not significantly different between the control and KA groups (Welch’s unpaired t-test, p = 0.3869, n = 5 control, n = 6 KA animals).

Having shown that intermediate gray matter SpIN loss correlates with the observed deficits, we wanted to investigate if there was any correlation between motoneuron loss and behavior. We counted the number of lower motoneurons at the injection epicenters and correlated them to the BBB score and inclined beam, and found no significant correlations (Fig 7A and 7B: BBB r = -0.2327 and p = 0.388; inclined beam r = -0.2338 and p = 0.226, Spearman’s correlation). Additionally, quantification of NeuN+ positive cells in lamina IX did not show significant differences between the two groups (Fig 7C).

Although KA targets gray matter primarily, we wanted to ensure we did not secondarily damage the white matter tracts, and therefore examined the area of white matter of the spinal cord cross-sectional area by eriochrome cyanine staining. Unlike classical contusion model analysis, we have three sites of possible damage and therefore examined each individual injection epicenter to confirm there were no white matter alterations. We did not observe any significant differences in white matter area at any given epicenter between the two groups (Fig 7D). Taken together, these results show that motoneurons and white matter were not significantly altered to contribute to the observed behavioral deficits.

### KA lesioned animals do not recover over time

**Fig 8:**
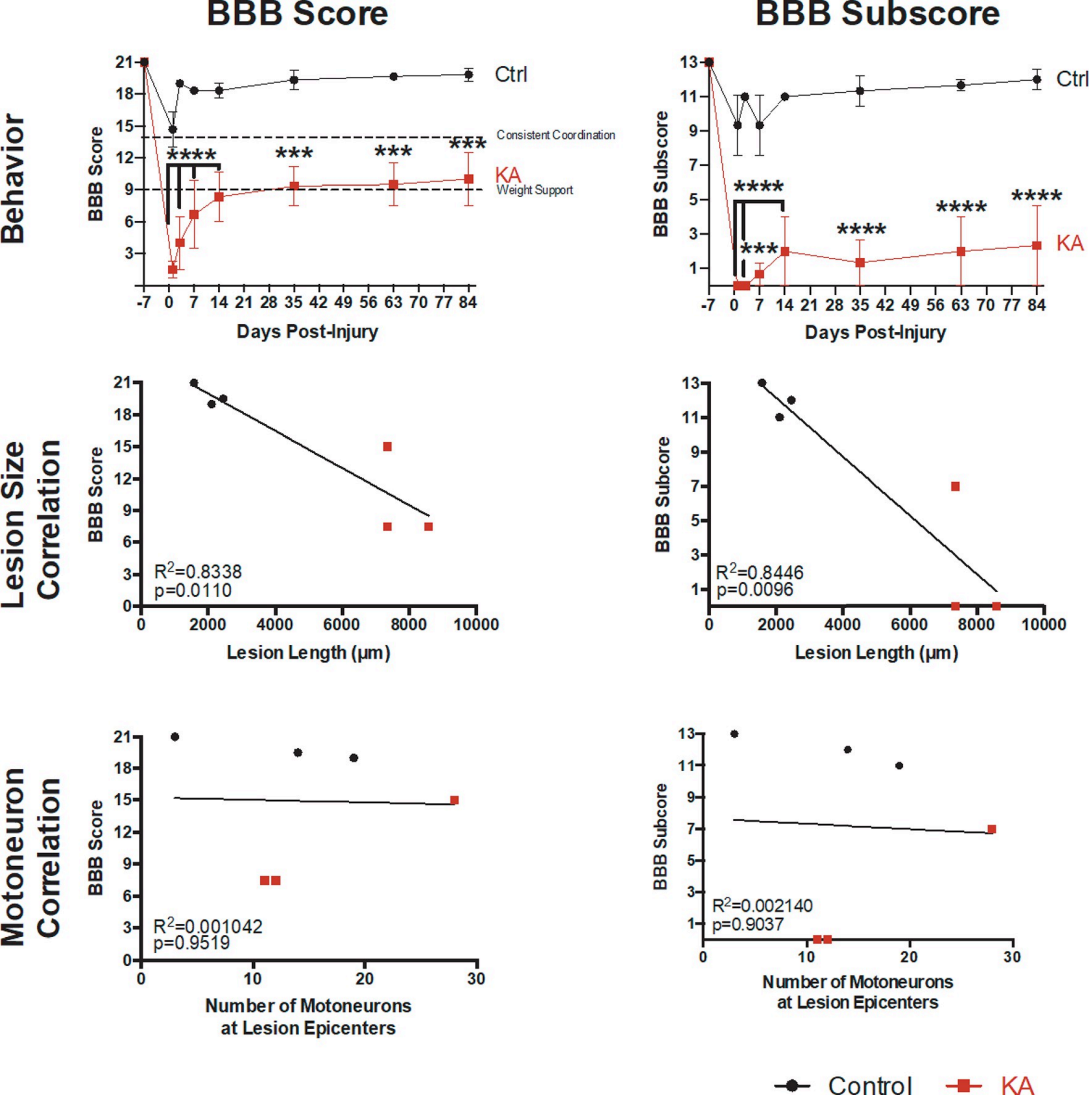
Analyzing the behavioral stability of the lesion after three months shows gross hindlimb and coordination deficits remain. (A-B) BBB score and subscore over a three-month period shows that gross hindlimb functional deficits remain (2-way ANOVA with Sidak’s post hoc test, BBB group p = 0.0062; BBB subscore group p = 0.0013). (C-D) BBB score and subscore correlate significantly to lesion length after three months (Linear Regression Analysis, BBB score p = 0.0110, R^2^ = 0.8338; BBB subscore p = 0.0096, R^2^ = 0.8446). (E-F) Remaining motoneurons at lesion epicenters do not correlate to behavioral performance after three months (Linear Regression Analysis BBB score p = 0.9516, R^2^ = 0.001042). For all data, n = 3 animals per group; *** p ≤ 0.001, **** p ≤ 0.0001.

Endogenous plasticity is thought to help regain function after thoracic SCI and should be evaluated over time. KA-lesioned rat behavioral performance three months post-injury reveal that deficits in gross hindlimb function and coordination remain (Fig 8A, 8B and S6 Fig). A closer look at the BBB score and subscore show that KA-injured rats have significant deficits in hindlimb function (Fig 8A and 8B: BBB score controls = 19.83 ± 0.60, KA = 10 ± 2.5; BBB subscore controls = 12 ± 0.58, KA = 2.33 ± 2.33). From the BBB score and subscore it appears that the KA lesioned animals did not recover. We found that while lesion length did correlate with the gross hindlimb function (Fig 8C and 8D: BBB score r = -0.8971, p = 0.0110; BBB subscore r = -0.8971, p = 0.0111, Spearman’s correlation), the motoneuron number at the injection epicenters did not (Fig 8E and 8F: BBB score r = -0.1160, p = 0.7778; BBB subscore r = -0.1160, p = 0.7778, Spearman’s correlation). This suggests that the targeting of the intermediate gray matter of the L2-L4 spinal cord leads to permanent locomotor deficits.

### Comprehensive behavioral classification

**Fig 9:**
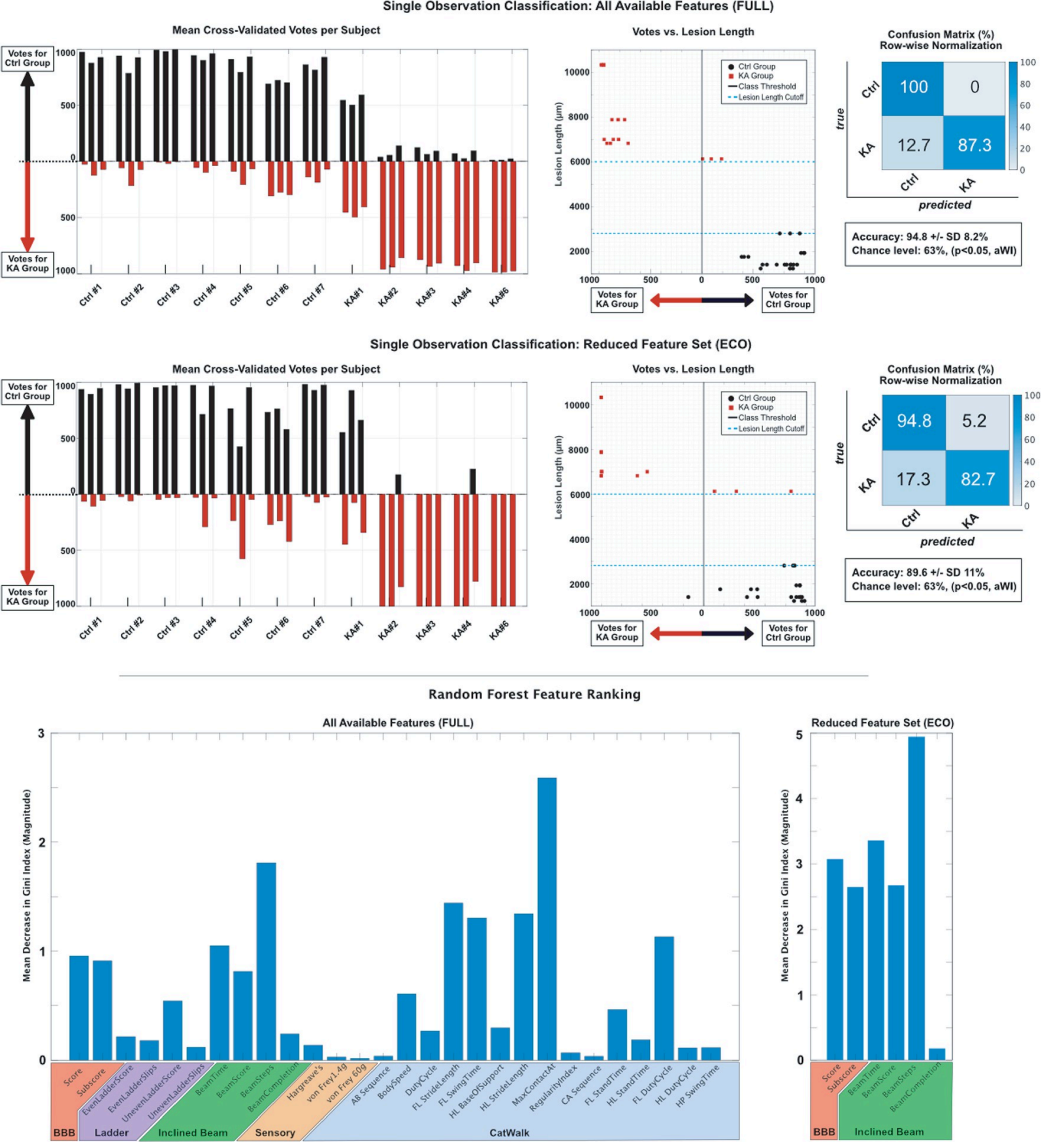
Behavioral classification of animals with the FULL and ECO models. (A,D) The left column shows the mean of the cross-validated votes (n = 1000) per observation per subject. For each animal, 3 observations were used (bar groups of three). Black bars indicate votes for the control group, red bars show votes for the KA group. Final class prediction was determined via majority vote. (B,E) The center column shows the relationship between votes (abscissa) and the post-hoc determined lesion length (ordinate). The class threshold is shown as horizontal line at point 0. The true class is shown in red for the KA group and in black for the control group. (C,F) The right column depicts the row-wise normalised confusion matrix for each classification approach as a percentage. The chance level was determined using an adjusted Wald Interval (aWI) at 63.0% (p<0.05). (G,H) Random Forest feature ranking according to the mean decrease in Gini Index for both FULL (G) and ECO (H) model. The higher the mean decrease, the more important the feature is considered to the classification model. N = 7 control and n = 5 KA animals.

To test the efficacy of experimental SCI therapies, it is important to evaluate discrete recovery performance through the combination of behavioral tests. In addition to carefully classifying groups at the end of the study, there is a need to provide exclusion criteria and equal sorting of variability between groups prior to treatment (after deficits have stabilized at two weeks). Therefore, using a Random Forest classification approach, we were able to develop two models that classify animals into their appropriate test groups using a combination of behavioral tests (Fig 9). For the comprehensive and sensitive FULL model, all available behavioral parameters mentioned above (BBB, horizontal ladder, inclined beam, sensory and CatWalk tests) were used with a mean accuracy of 94.8 ± SD 8.2% (chance level 63%, p<0.05, adjusted Wald Interval) reached. The confusion matrix indicates that 12.7% of the KA observations were misclassified as control (Type 2 error). The mean of the normalized votes indicates that 11 out of 12 animals were appropriately classified animals into either the control or KA groups (Fig 9A and 9C).

As most behavioral analysis is highly time intensive, we also strove to develop a sensitive classification model that relied upon quick functional evaluation. A fast early classification approach used a reduced feature set (ECO) focusing only on the BBB and inclined beam behavioral tests and reached a mean accuracy of 89.6 ± SD 11%. The confusion matrix shows a misclassification rate of 5.2% (Type 1 error) for all control observations and 17.3% for all KA observations (Type 2 error) (Fig 9F). This is also reflected in the mean of the cross-validated votes (aggregated decisions of the ensembled decision trees) per subject: 11 out of 12 animals were also appropriately classified (Fig 9D and 9E). This Type 2 error seen in both models is understandable given the location and lesion extent of this animal (KA#1) compared to the rest of the animals, confirming our previous observations.

To validate the endpoint lesion length in the rostro-caudal axis as an exclusion criteria, we correlated the votes per trial with the corresponding animal lesion lengths and found significant correlations (Fig 9B and 9E: FULL model r = -0.7395 and p <0.007, ECO model r = -0.8916 and p <0.0001, Spearman’s correlation). The blue dotted lines indicate histologically determined cutoffs for the control and KA lesion lengths (2800μm and 6000μm, respectively) and further shows that the misclassified animal (KA#1) is the closest to this cutoff (Fig 9B and 9E). Together, these findings highlight the sensitivity and accuracy of the FULL and ECO models.

To determine which behavioral tests were most predictive and important for correct classification, a full ranking of the behavioral parameters was performed for both models and presented as the mean decrease of the Gini Index; the greater the decrease, the more discriminative information the behavioral test provides. For the FULL classification model, the highest rankings are achieved by the selected CatWalk parameters, however, all parameters obtained from the BBB and the inclined beam also showed consistent high rankings (Fig 9G). For the ECO model, rankings were all above 2.5 except for inclined beam completion (Fig 9H). Using these two models, combined behavioral performance and potential recovery after injury can be compared and evaluated both during the ongoing experiment with the ECO model, as well as post time-intensive analysis of all behavioral tests with the FULL model.

## Discussion

Targeted KA lesions to the intermediate gray matter at spinal levels L2-L4 induced profound behavioral deficits related to locomotion. Damage to this area, which includes regulation of lower motoneurons leads not only to gross motor deficits (BBB score), but rhythmic and skilled walking (even and uneven horizontal ladders), coordination (BBB subscore), balance (inclined beam) and gait deficits (CatWalk), as well. We have shown that this lesion damages the intermediate gray matter with the dorsal horn (laminae I-IV) remaining relatively undisturbed (no significant KA-induced sensory deficits and no neuronal quantification differences after two weeks). We did not find alterations in motoneuron numbers that would indicate a relationship with observed behavioral deficits. KA targeted specifically gray matter as we did not observe white matter loss. Neuronal loss in laminae V-VIII correlated with coordination and balance deficits and provided further insight into the functional role of the IN populations spanning the spinal L2-L4 region. After three months, KA rats did not regain any lost sensorimotor function. This work indicates: one, the functional deficits of such an injury in this region; two, such an injury cannot be naturally compensated for; and three, that additional therapies must be considered for functional recovery.

### KA lesion targets local and propriospinal INs in intermediate gray matter and are crucial for locomotion/coordination

In laminae V-VIII of the intermediate gray matter reside both local SpINs and propriospinal INs which have been shown to play various regulatory roles in locomotion. Previous studies have found that treadmill walking activated spinal levels T13-L6 in adult rats, with significantly more cFos+ neurons in laminae IV, V and VII in trained versus untrained rats [16]. Furthermore, activity profiling of cFos+ SpINs in the mouse lumbar spinal cord following rotarod training revealed that many SpINs specific to motor activity (ie not active during baseline or painful formalin stimulation) are located in the intermediate gray matter laminae, including Gad2+ cells, NF1b+ cells and V2a neurons [2]. Therefore, it was likely this region would be integral to locomotion, however it remained to be seen what type of deficits would be displayed when the spinal excitatory/inhibitory IN ratio was shifted. We have found that collective damage to this area creates significant and lasting locomotor deficits that particularly affect coordination.

KA is an excitotoxin that has some selectivity for INs over motoneurons [44, 45]. We have confirmed that the region of laminae V-VIII is indeed damaged with our lesion. We observed that the number of motoneurons did not correlate with behavioral deficits nor were significant sensory differences found between the control and KA groups. Neuronal damage to the dorsal horn can evoke pain-related behavior in mice [9] and animals with pain affecting their hindlimbs display an altered gait including in duty cycle and stride length parameters [10, 11]. The fact that there are no significant differences in mechanical or thermal sensitivity or neuronal quantification of laminae I-IV, validates that KA did not affect the dorsal horn and impact our gait-related behavioral findings. Further, white matter tracts were not targeted by KA and may signify descending control of lower motoneurons could have remained intact [46–48] and should be further examined. These findings, therefore, indicate the specificity of our lesion and that damage to the SpINs in the intermediate gray matter which provide regulatory input to motoneurons resulted in the observed deficits.

### Lesioned intermediate SpINs spanning L2-L4 and at least 6mm in length produce significant behavioral deficits

After behavioral analysis, we observed that the most predictive factors of a properly lesioned KA-animal were lesion length in the rostro-caudal axis spanning spinal L2-L4 and neuronal loss in laminae V-VIII. We determined that KA-lesioned animals needed to have a minimum intermediate gray matter lesion length of at least 6mm in the rostro-caudal length to produce consistent behavioral deficits using our behavioral tests. In lesions shorter than 6mm, SpINs and premotor circuitry in other spinal segments can compensate for the neuronal loss, resulting in very minor or no behavioral deficits (these animals were excluded from the analysis). In lesions greater than 6mm, we observed significant correlation with locomotor function after two weeks. The average lumbar cord (L1-L6) of the adult *Rattus norvegicus* spans approximately 18mm. Spinal levels L2-L4 measure approximately 10mm, of which each spinal segment spanned slightly over 3mm [49]. These findings suggest that the lesion length must span at least two spinal levels. For context regarding the SpIN and premotor targeted circuitry, it should be noted that hindlimb muscles are innervated by motoneurons spanning several lumbar spinal levels [50–52]. The muscles vastus medialis, vastus lateralis, gracillis, tibialis anterior, biceps femoris muscles are innervated by motoneurons from at least two spinal levels in spinal L2-L4 [53, 54]. Therefore, the targeted regulatory circuitry of the motoneurons must also span a similar range. Although the lesions reported here are slightly longer than what has been previously published (3.4mm or 5.5mm), those previous lesions used 1.5μl of 5mM or 2.5mM KA per injection to produce larger gray matter lesions with more neuronal loss and also severely damaged dorsal horns [8].

While lesion length in the rostro-caudal axis plays an important role, it does not appear to be the only determining factor. For example, some animals have the same lesion extent, but varying behavioral deficits. We believe this may be due to the severity of the injury at given levels. The Random Forest classification and neuronal quantification highlight that this damage must include spinal level L2 to observe deficits in coordination and balance. This is most clearly demonstrated by animal KA #1, with a lesion length longer than 6mm (6125μm), exhibiting neuronal loss primarily in L3 and L4. The Random Forest classification of animal KA #1 produced a Type 2 error that lead to misclassification as a control due to its behavioral performance. All other animals with greater lesion lengths and neuronal loss were properly classified. In the long-term group, there were two animals with the same lesion length (7350μm), however one had weight support and the other did not. Neuronal analysis revealed that the animal without weight support (LT KA #2) had almost twice the amount of neuronal loss in spinal L4 in comparison to the animal with weight support (LT KA #1). This indicates that spinal L2 and L4 may be integral to but not solely responsible for the observed behavioral loss. These findings for spinal L2 support what has been previously published regarding circuitry critical to locomotion residing at this level in both the previously mentioned murine KA models and human models [7, 8, 55] and further illustrates the contribution of specifically the intermediate gray matter in locomotion.

### CatWalk gait analysis reveals deficits in rhythm and pattern generation

The CatWalk measures dynamic and static gait parameters. We initially focused on the pLDA score which draws on nine parameters that characterize contusion and dorsal hemisection SCI models. While both SCI models reside in the thoracic region, they target descending tracts which connect with lumbar premotor circuitry at varying degrees. Such differences were not categorized by the BBB gross hindlimb score but were highlighted by the pLDA score [27]. Our Gini Index results confirm that the CatWalk pLDA and subsequent parameters are highly predicative and that damage to gray matter in this particular region significantly alters gait.

CatWalk analysis revealed KA animals had rhythmic deficits (longer stand time and shorter swing time) however surprisingly these differences were primarily found in the forelimbs, not the hindlimbs. Furthermore, the Gini Index found several CatWalk forelimb parameters to be highly discriminant despite the lesion residing in the lumbar cord. Long propriospinal INs located in laminae VII-VIII connect the cervical and lumbar central pattern generator (CPG) networks (including in spinal levels C6-C8 and L1-L3) [40, 42, 55–58]. This lesion model created neuronal loss in laminae V-VIII, a region where several ascending propriospinal somas reside and descending propriospinal INs terminate [13, 59]. Therefore, while these changes could be a compensatory mechanism [60], we hypothesize this may be due to propriospinal damage uncoupling the fore-and hindlimbs.

In addition to gait rhythm changes, we also saw significant differences in pattern generation. The regularity index (RI) measures correctly sequenced footsteps and is used to analyze recovery in mild to moderate injuries and coordination [61–63]. While KA-animals have a significantly lower RI in comparison to the controls, the RI remains above 90% which is still relatively high given the amount of neuronal loss. However, we would argue that a single parameter is not the defining factor of gait/coordination, but a combinatorial pattern of parameters and tests provides a more comprehensive picture, as we have seen with our pLDA analysis and Random Forest classification approaches.

We observed that KA-lesioned animals have significantly higher CA step sequences, as opposed to the more commonly seen AB sequence. Previous papers have reported speed can influence step sequence in mice and rats and that a faster gait corresponds to AB sequence [64, 65]. As our KA-lesioned rats have significantly slower body speed, this change in gait pattern is not surprising. Additionally, the step sequence change observed in KA animals could further be indicative of either long or short propriospinal damage. KA-lesioned animals have significantly higher CA step sequences, as opposed to the more commonly seen AB sequence which suggests interlimb coordination deficits [66] a characteristic also observed after long descending propriospinal ablation or stimulation [15, 67]. A previous study has shown that an increase in cruciate stepping was also observed after silencing descending spinal L2 short propriospinal INs which project to L5. This change in gait could therefore also be due to L2-L5 IN loss, as they are found in the intermediate gray matter (primarily laminae V-VIII) [41], where our KA lesion also resides.

Together, these results show that KA-lesioned rats have deficits in gait rhythm and pattern parameters and further suggest propriospinal IN damage.

### Damage to the lumbar spinal enlargement is permanent and cannot be compensated

For this study, we were interested in determining how essential the intermediate gray matter of the lumbar cord is for locomotion and if redundant pathways or other neurons can compensate for SpIN loss. Previous studies have shown that following SCI, propriospinal INs have the capacity to sprout and form new synaptic connections, with the potential to contribute to functional gains [61, 68–73]. The behavioral results of this study suggest that when lumbar spinal and propriospinal INs are lost, there are no other intrinsic mechanisms that can recover lost motor function. Hindlimb deficits level off after two weeks and remain stable even after three months. In agreement, the two animals that did not have weight support after two weeks did not regain weight support at three months. Therefore, this model is well suited to investigate the efficacy of neurorestorative therapies after SCI in the lumbar spinal enlargements.

### Random Forest modeling determines classification prior to histological analysis

Experimental SCI models often have exclusion criteria to determine if the surgery was carried out properly, such as the displacement curve for contusion models [74–76]. There is no such parameter available for this KA model, and lesion length and neuronal loss can only be determined post-mortem after time-intensive analysis. We aimed to create a mathematical model based on behavior that can be used to verify whether animals were properly lesioned. While Gini Index findings of the FULL model indicated that the select CatWalk parameters are the most predicative for proper classification, this apparatus is expensive and not easily available to all researchers. We therefore focused on the BBB and inclined beam behavioral tests for the ECO model, both of which use equipment that is more generally accessible, cost-effective and scoring can be completed quickly. By limiting the mathematical model to these two assessments, we slightly decrease reliability but keep a strong classification rate as observed in the ECO model (FULL model: 94.8% accuracy and ECO model: 89.6% accuracy). Our recommendation is for the ECO model to be used as an early exclusion criterion and for balancing group differences prior to intervention. However, this intervention would need to start after the two-week period when the behavioral data is collected and analyzed, therefore it lends itself more effectively to cellular replacement therapies. The FULL model provides a more sensitive behavioral evaluation than a single test alone and can be used post-experiment to analyze intervention effects.

## Conclusions

The results of this study highlight the role intermediate gray matter SpINs play in the lumbar enlargement. Furthermore, they do not seem to be compensated for by spontaneous remodeling. Previous studies have compared contusion and KA injuries in both the thoracic and lumbar cord and have shown that the lumbar gray matter plays a greater role in locomotion than the thoracic gray matter [8]. Our findings show that damage to the lumbar intermediate gray matter creates lasting deficits beyond gross hindlimb function and particularly affects coordination. Therefore, while many SCI treatments focus on regeneration of host neurons, primarily of the white matter tracts, other treatments such as gray matter replacement therapies are required for injuries to the lumbar enlargement. This new SCI model with fully characterized behavioral deficits and sensitive neuronal quantification and classification models will help carefully evaluate the potential of a cellular replacement therapy and aid future SCI treatment options.

## Supporting information

S1 Table Summary of animal experiments and parameters. (PDF)

S2 Table Overview of behavioral tests. (PDF)

S3 Table Feature extraction and observation generation for the FULL model. (PDF)

S4 Table Feature extraction and observation generation for the ECO model. (PDF)

S1 Fig Experimental timeline of short-term behavioral testing experiment. First, animals underwent habituation and baseline testing during the week prior to the surgery. Following KA injections, the BBB test was performed 1, 3 and 7 days post-injury. One and two weeks after injury, the inclined beam, ladder, von Frey and Hargreave’s tests were additionally performed. Animals underwent CatWalk habituation and testing on days 19–20 after injury.
(PDF)

S2 Fig Experimental timeline of long-term behavioral testing experiment. First, animals underwent habituation and baseline testing during the week prior to the surgery. Following KA injections, the BBB test was performed 1, 3, 7, 14, 35, 63, 84, 89 days post-injury. One, two and three months after injury, the inclined beam, ladder, von Frey and Hargreave’s tests were additionally performed. Animals underwent CatWalk habituation and testing on days 89–90 after injury.
(PDF)

S3 Fig Image acquisition and analysis workflow designed for neuronal quantification. First, Z-stack tiles of coronal slices stained with a NeuN antibody were acquired using a confocal XT1000 microscope with the 10x magnification objective. The tiles were stitched in ImageJ/Fiji and a spinal cord atlas overlay was registered over the maximum intensity projection using the BUnwarpJ ImageJ/Fiji plugin. Once the correct spinal levels and ROIs were determined (laminas V-VIII), the ilastik pixel classification workflow was trained, and the output foreground probability map used as an input in cellpose to 3D segment the nuclei (cellpose, nuclei pretrained model). The 3D labeled images were visualized in arivis Vision4D and segmentation mistakes were manually corrected. The neuronal counts were normalized by ROI volume.
(PDF)

S4 Fig Rhythmic component in gait is significantly affected in KA-injured animals two weeks post-injury. Average swing time, stand time and duty cycle were significantly different for the forelimbs but not for the hindlimbs (Welch’s unpaired t-test (A) p = 0.022; (B) p = 0.2939; (C) p = 0.0441; (D) p = 0.6858; (E) p = 0.0031; (F) = 0.6807). N = 7 control and n = 5 KA animals; * p ≤ 0.05, ** p ≤ 0.01.
(PDF)

S5 Fig Representative heatmap comparing control and KA NeuN-positive cells in laminae V-VIII in spinal levels L2-L4 to behavioral performance. All neuronal values and behavioral performances are normalized to the highest control value; the lesion length is normalized to the largest lesion extent (KA #5). All values are shown as a percentage (0% in violet, 100% in yellow).
(PDF)

S6 Fig Representative heatmap comparing control and KA NeuN-positive cells in laminae V-VIII in spinal levels L2-L4 to behavioral performance for animals in the long-term experiment. All neuronal values and behavioral performances are normalized to the average controls; the lesion length is normalized to the largest lesion extent (LT KA #3). All values are shown as a percentage (0% in violet, 100% in yellow).
(PDF)

S1 Video Representative animal performance on even horizontal ladder, uneven horizontal ladder and inclined beam. (MP4)
