# Supplementary material for: Intermediate gray matter interneurons in the lumbar spinal cord play a critical and necessary role in coordinated locomotion
Source: PLoS One. 2023 Oct 31;18(10):e0291740. doi: 10.1371/journal.pone.0291740 (PMC10617729; doi:10.1371/journal.pone.0291740)
Supplement: S1 Table — (PDF) [file pone.0291740.s001.pdf]

Supporting Table 1. Summary of animal experiments and parameters.

Experiment	# Animals (Control/KA)	Injection Parameters	Time	Behavioral Tests
**Group 1 (Pilot Experiments)**	12 rats, 3 excluded Total: 9 (4/5)	Laminectomy T12 1-2 bilateral KA injections	2 weeks	BBB, uneven horizontal Ladder
**Group 2**	21 rats, 7 excluded Total: 14 (7/7)	Laminectomy T13, 3 bilateral KA injections	2 weeks	BBB, even and uneven horizontal ladder, inclined beam, von Frey, Hargreave's, CatWalk
**Group 3**	9 rats, 3 excluded Total: 6 (3/3)	Laminectomy T13, 3 bilateral KA injections	3 months	BBB, even and uneven horizontal ladder, inclined beam, von Frey, Hargreave's, CatWalk
