# Supplementary material for: Intermediate gray matter interneurons in the lumbar spinal cord play a critical and necessary role in coordinated locomotion
Source: PLoS One. 2023 Oct 31;18(10):e0291740. doi: 10.1371/journal.pone.0291740 (PMC10617729; doi:10.1371/journal.pone.0291740)
Supplement: S2 Table — (PDF) [file pone.0291740.s002.pdf]

Supporting Table 2. Overview of behavioral tests.

Behavioral Test	Function
BBB	Gross hindlimb function
Even Horizontal Ladder	Rhythmic walking
Uneven Horizontal Ladder	Sensorimotor coordination
Inclined Beam	Balance and Coordination
Von Frey	Static mechanical sensitivity
Hargreave's	Thermal sensitivity
CatWalk	Gait
