# Supplementary material for: Intermediate gray matter interneurons in the lumbar spinal cord play a critical and necessary role in coordinated locomotion
Source: PLoS One. 2023 Oct 31;18(10):e0291740. doi: 10.1371/journal.pone.0291740 (PMC10617729; doi:10.1371/journal.pone.0291740)
Supplement: S3 Table — (PDF) [file pone.0291740.s003.pdf]

Supporting Table 3. Feature extraction and observation generation for the FULL model.

#	Feature Type	# Repetitions	Method
1	'BBBScore'	1	Duplicate for each observation per animal
2	'BBBSubscore'	1	Duplicate for each observation per animal
3	'EvenLadderScore'	[5 1]	First 3 repetitions
4	'EvenLadderSlips'	[5 1]	First 3 repetitions
5	'UnevenLadderScore'	[5 1]	First 3 repetitions
6	'UnevenLadderSlips'	[5 1]	First 3 repetitions
7	'ICBeamTime'	[3 1]	First 3 repetitions
8	'ICBeamScore'	[3 1]	First 3 repetitions
9	'ICBeamSteps'	[3 1]	First 3 repetitions
10	'ICBeamCompletions'	[3 1]	First 3 repetitions
11	'Hargreaves'	[4 1]	First 3 repetitions
12	'Frey1_4'	[5 1]	First 3 repetitions
13	'Frey60'	[5 1]	First 3 repetitions
14	'CWABSeq'	[5 1]	First 3 repetitions
15	'CWBodySpeed'	[5 1]	First 3 repetitions
16	'CWDutyCycle'	[5 1]	First 3 repetitions
17	'CWFLStrideLength'	[5 1]	First 3 repetitions
18	'CWFLSwingTime'	[5 1]	First 3 repetitions
19	'CWHLBaseOfSupport'	[5 1]	First 3 repetitions
20	'CatwalkHLStrideLength'	[5 1]	First 3 repetitions
21	'CWMaxContactAt'	[5 1]	First 3 repetitions
22	'CWRegIdx'	[5 1]	First 3 repetitions
23	'CWCASequence'	[5 1]	First 3 repetitions
24	'CWFLStandTime'	[5 1]	First 3 repetitions
25	'CWHLStandTime'	[5 1]	First 3 repetitions
26	'CWFLDutyCycle'	[5 1]	First 3 repetitions
27	'CWHLDutyCycle'	[5 1]	First 3 repetitions
28	'CWHLSwingTime'	[5 1]	First 3 repetitions
