# Supplementary material for: Intermediate gray matter interneurons in the lumbar spinal cord play a critical and necessary role in coordinated locomotion
Source: PLoS One. 2023 Oct 31;18(10):e0291740. doi: 10.1371/journal.pone.0291740 (PMC10617729; doi:10.1371/journal.pone.0291740)
Supplement: S4 Table — (PDF) [file pone.0291740.s004.pdf]

Supporting Table 4. Feature extraction and observation generation for the ECO model.

#	Feature Type	# Repetitions	Method
1	'BBBScore'	1	Duplicate for each observation per animal
2	'BBBSubscore'	1	Duplicate for each observation per animal
3	'ICBeamTime'	[3 1]	First 3 repetitions
4	'ICBeamScore'	[3 1]	First 3 repetitions
5	'ICBeamSteps'	[3 1]	First 3 repetitions
6	'ICBeamCompletions'	[3 1]	First 3 repetitions
