# Supplementary material for: Intermediate gray matter interneurons in the lumbar spinal cord play a critical and necessary role in coordinated locomotion
Source: PLoS One. 2023 Oct 31;18(10):e0291740. doi: 10.1371/journal.pone.0291740 (PMC10617729; doi:10.1371/journal.pone.0291740)
Supplement: S1 Fig — First, animals underwent habituation and baseline testing during the week prior to the surgery. Following KA injections, the BBB test was performed 1, 3 and 7 days post-injury. One and two weeks after injury, the inclined beam, ladder, von Frey and Hargreave’s tests were additionally performed. Animals underwent CatWalk habituation and testing on days 19–20 after injury. (PDF) [file pone.0291740.s005.pdf]

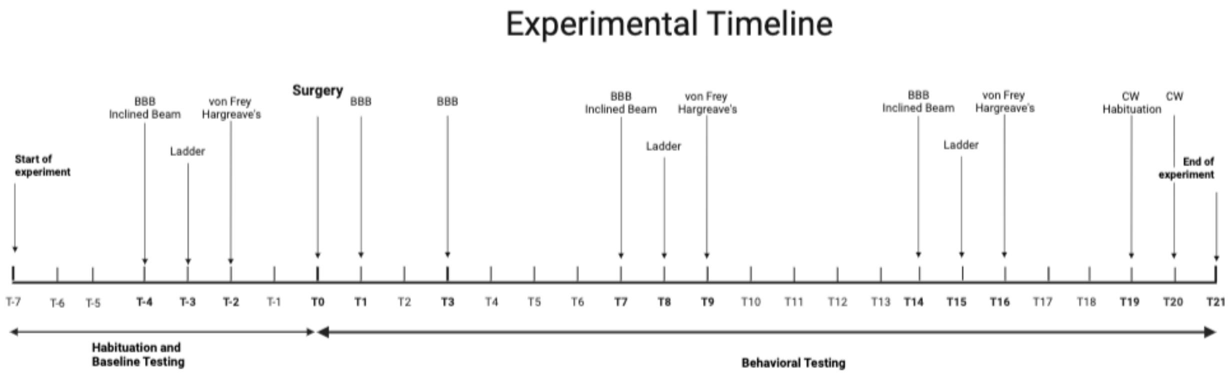

Supporting Figure 1. Experimental timeline of short-term behavioral testing experiment. First, animals underwent habituation and baseline testing during the week prior to the surgery. Following KA injections, the BBB test was performed 1, 3 and 7 days post-injury. One and two weeks after injury, the inclined beam, ladder, von Frey and Hargreave's tests were additionally performed. Animals underwent CatWalk habituation and testing on days 19-20 after injury.
