# Supplementary material for: Intermediate gray matter interneurons in the lumbar spinal cord play a critical and necessary role in coordinated locomotion
Source: PLoS One. 2023 Oct 31;18(10):e0291740. doi: 10.1371/journal.pone.0291740 (PMC10617729; doi:10.1371/journal.pone.0291740)
Supplement: S2 Fig — First, animals underwent habituation and baseline testing during the week prior to the surgery. Following KA injections, the BBB test was performed 1, 3, 7, 14, 35, 63, 84, 89 days post-injury. One, two and three months after injury, the inclined beam, ladder, von Frey and Hargreave’s tests were additionally performed. Animals underwent CatWalk habituation and testing on days 89–90 after injury. (PDF) [file pone.0291740.s006.pdf]

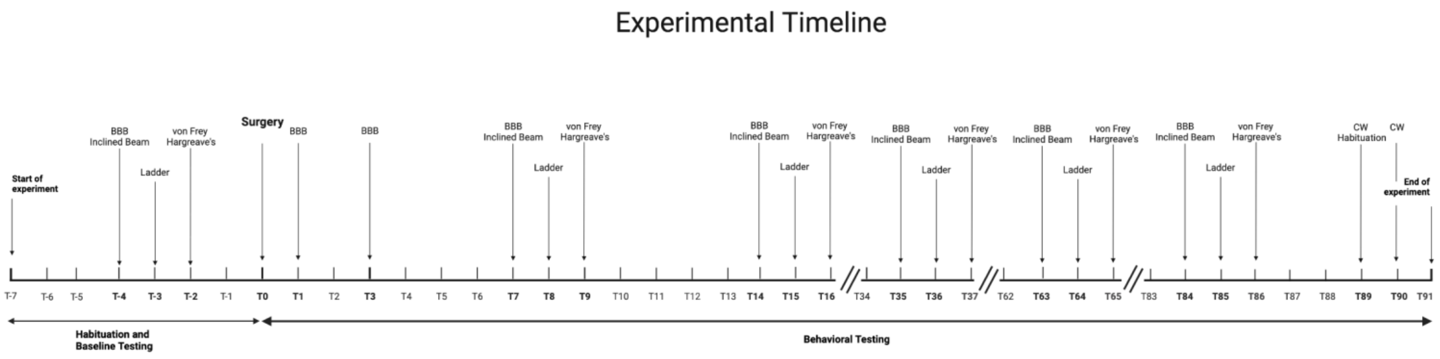

Supporting Figure 2. Experimental timeline of long-term behavioral testing experiment. First, animals underwent habituation and baseline testing during the week prior to the surgery. Following KA injections, the BBB test was performed 1, 3, 7, 14, 35, 63, 84, 89 days post-injury. One, two and three months after injury, the inclined beam, ladder, von Frey and Hargreave’s tests were additionally performed. Animals underwent CatWalk habituation and testing on days 89-90 after injury.
