# Supplementary material for: Intermediate gray matter interneurons in the lumbar spinal cord play a critical and necessary role in coordinated locomotion
Source: PLoS One. 2023 Oct 31;18(10):e0291740. doi: 10.1371/journal.pone.0291740 (PMC10617729; doi:10.1371/journal.pone.0291740)
Supplement: S3 Fig — First, Z-stack tiles of coronal slices stained with a NeuN antibody were acquired using a confocal XT1000 microscope with the 10x magnification objective. The tiles were stitched in ImageJ/Fiji and a spinal cord atlas overlay was registered over the maximum intensity projection using the BUnwarpJ ImageJ/Fiji plugin. Once the correct spinal levels and ROIs were determined (laminas V-VIII), the ilastik pixel classification workflow was trained, and the output foreground probability map used as an input in cellpose to 3D segment the nuclei (cellpose, nuclei pretrained model). The 3D labeled images were visualized in arivis Vision4D and segmentation mistakes were manually corrected. The neuronal counts were normalized by ROI volume. (PDF) [file pone.0291740.s007.pdf]

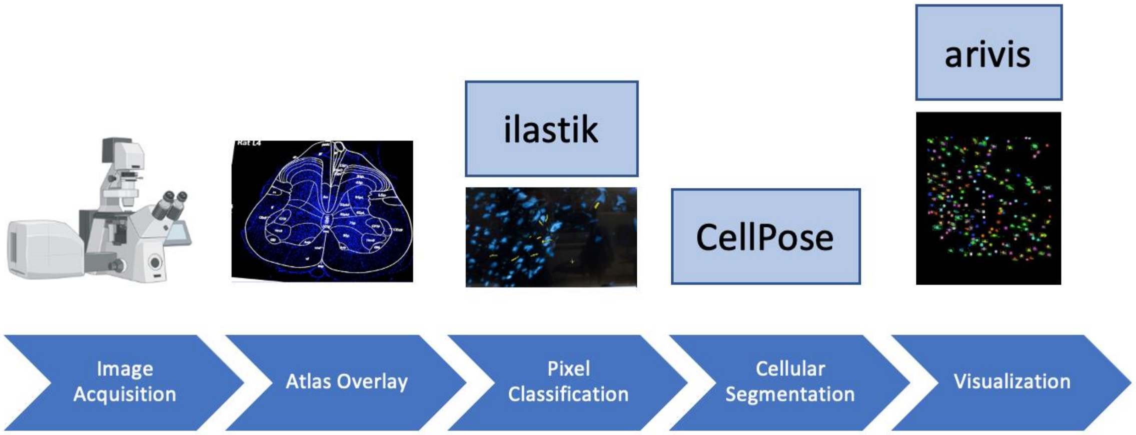

Supporting Figure 3. Image acquisition and analysis workflow designed for neuronal quantification. First, Z-stack tiles of coronal slices stained with a NeuN antibody were acquired using a confocal XT1000 microscope with the 10x magnification objective. The tiles were stitched in ImageJ/Fiji and a spinal cord atlas overlay was registered over the maximum intensity projection using the BUnwarpJ ImageJ/Fiji plugin. Once the correct spinal levels and ROIs were determined (laminae I-IV, V-VIII, IX), the ilastik pixel classification workflow was trained, and the output foreground probability map used as an input in cellpose to 3D segment the nuclei (cellpose, nuclei pretrained model). The 3D labeled images were visualized in arivis Vision4D and segmentation mistakes were manually corrected. The neuronal counts were normalized by ROI volume.
