# Supplementary material for: Intermediate gray matter interneurons in the lumbar spinal cord play a critical and necessary role in coordinated locomotion
Source: PLoS One. 2023 Oct 31;18(10):e0291740. doi: 10.1371/journal.pone.0291740 (PMC10617729; doi:10.1371/journal.pone.0291740)
Supplement: S4 Fig — Average swing time, stand time and duty cycle were significantly different for the forelimbs but not for the hindlimbs (Welch’s unpaired t-test (A) p = 0.022; (B) p = 0.2939; (C) p = 0.0441; (D) p = 0.6858; (E) p = 0.0031; (F) = 0.6807). N = 7 control and n = 5 KA animals; * p ≤ 0.05, ** p ≤ 0.01. (PDF) [file pone.0291740.s008.pdf]

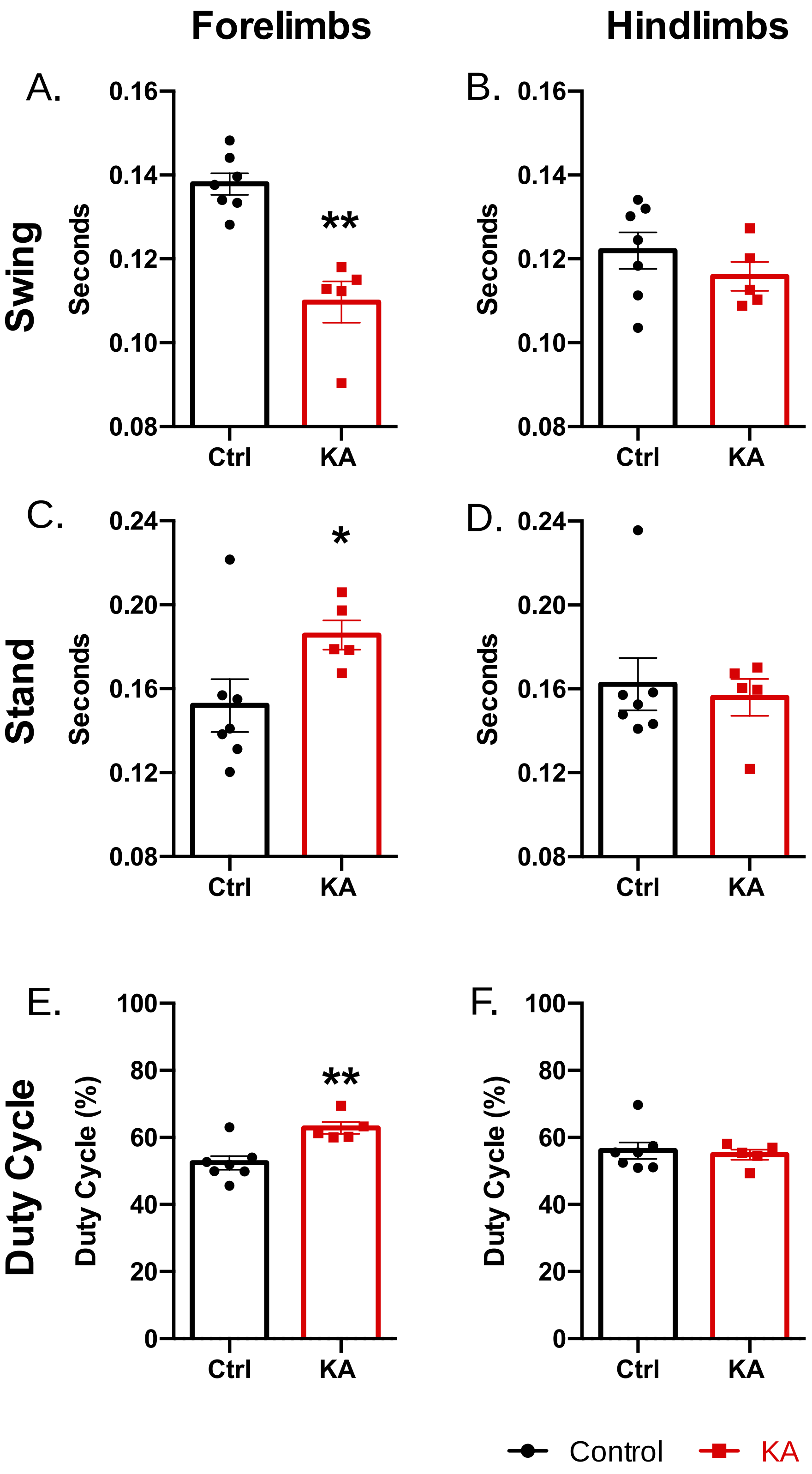

Supporting Figure 4. Rhythmic component in gait is significantly affected in KA-injured animals two weeks post-injury. ***A-F*** Average swing time, stand time and duty cycle were significantly different for the forelimbs but not for the hindlimbs (Welch's unpaired t-test ***A,*** p = 0.022; ***B,*** p = 0.2939; ***C,*** p = 0.0441; ***D,*** p = 0.6858; ***E,*** p = 0.0031; ***F,*** = 0.6807). N = 7 control and n = 5 KA animals; * p ≤ 0.05, ** p ≤ 0.01.
