# Supplementary material for: Intermediate gray matter interneurons in the lumbar spinal cord play a critical and necessary role in coordinated locomotion
Source: PLoS One. 2023 Oct 31;18(10):e0291740. doi: 10.1371/journal.pone.0291740 (PMC10617729; doi:10.1371/journal.pone.0291740)
Supplement: S6 Fig — All neuronal values and behavioral performances are normalized to the average controls; the lesion length is normalized to the largest lesion extent (LT KA #3). All values are shown as a percentage (0% in violet, 100% in yellow). (PDF) [file pone.0291740.s010.pdf]

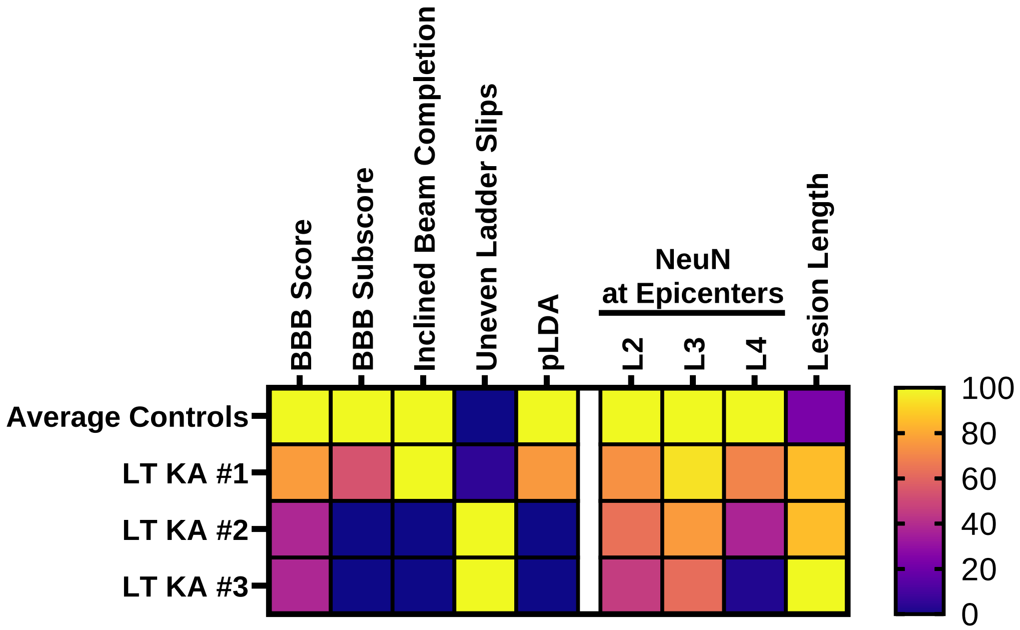

Supporting Figure 6. Representative heatmap comparing control and KA NeuN-positive cells in laminae V-VIII in spinal levels L2-L4 to behavioral performance for animals in the long-term experiment. All neuronal values and behavioral performances are normalized to the average controls; the lesion size is normalized to the largest lesion length (LT KA #3). All values are shown as a percentage (0% in violet, 100% in yellow).
